# The Impact of the COVID-19 Pandemic on Weight Loss and Quality of Life One Year Post Metabolic Bariatric Surgery

**DOI:** 10.1007/s11695-026-08515-6

**Published:** 2026-03-09

**Authors:** Michael Hanselmann, Sophie Merzweiler, Renée Stark, Ann-Cathrin Koschker, Carsten Klinger, Miljana Vladimirov, Florian Seyfried, Bettina Zippel-Schultz, Martin Fassnacht, Michael Laxy

**Affiliations:** 1 School of Medicine and Health, Technical University of Munich, Munich, Germany; 2 Munich Center of Health Economics and Policy, Munich, Germany; 3 Department of Internal Medicine I, University of Würzburg, Wurzburg, Germany; 4 Interdisciplinary Obesity Centre, University of Würzburg, Wurzburg, Germany; 5 German society for general and visceral surgery (DGAV), Berlin, Germany; 6 Medical School and University Medical Center Ostwestfalen-Lippe, Bielefeld University, Bielefeld, Germany; 7 Department of General, Visceral, Transplant, Vascular, and Pediatric Surgery, University of Würzburg, Wurzburg, Germany; 8 Department Innovation in Health Care, German Foundation for the Chronically Ill, Berlin, Germany

## Abstract

**Introduction:**

Postoperative follow-up care after metabolic bariatric surgery (MBS) is considered essential to achieve optimal outcomes. These follow-up care pathways were partially disrupted during the COVID-19 pandemic. Our objective was to investigate whether the COVID-19 pandemic and in particular the first (spring 2020) and the second wave (winter 2020/2021) had an impact on short-term outcomes of MBS in Germany.

**Methods:**

We analyzed data of 5,859 patients from 154 bariatric centers across Germany. Our predefined endpoints were percentage total weight loss (%TWL), bariatric quality of life (BQL), prevalence of type 2 diabetes (T2D), hypertension 1-year post-surgery and follow-up participation 3 months and 1 year post surgery. We compared these outcomes for MBS patients affected by the COVID-19 pandemic between 2019-12-01 and 2020-02-29 (COVID-19 group) with a corresponding control group that was not affected by the pandemic (No-COVID-19 group; surgery: 2018-12-01–2019-02-28).

**Results:**

Follow-up participation was significantly lower in the COVID-19 group (3-month: 75.3% vs. 82.7%, OR = 0.62; 1-year: 63.7% vs. 71.3%, OR = 0.69; both *p* < 0.001). Among patients attending the 1-year follow-up, %TWL, T2D, and hypertension rates did not differ significantly. However, BQL was slightly lower in the COVID-19 group (–0.14; *p* < 0.001).

**Conclusions:**

During the first and second wave of the COVID-19 pandemic frequency of follow-up visits after MBS was reduced in Germany. For those who participated, the quality of life was slightly reduced while all other outcomes were comparable. Our findings contribute to a better understanding of the impact of the COVID-19 pandemic - and lockdown measures in general – on short-term MBS outcomes.

**Supplementary Information:**

The online version contains supplementary material available at 10.1007/s11695-026-08515-6.

## Key Points

To date, evidence on the impact of the COVID-19 pandemic on short-term outcomes of metabolic and bariatric surgery (MBS) remains inconclusive.

In this study, we analyzed a large dataset from a German registry to assess the effect of the COVID-19 pandemic on short-term outcomes after MBS.

We found no effect on weight loss, a small but significant effect on quality of life, and a large, significant effect on participation in follow-up visits.

Our findings underscore the importance of ensuring continuity of care during future public health crises.

**Supplementary Information:**

The online version contains supplementary material available at 10.1007/s11695-026-08515-6.

## Introduction

In March 2020, the WHO declared COVID-19 a pandemic. Social distancing measures across Europe disrupted healthcare systems and may have particularly affected MBS patients. Several studies suggest that the emotional instability resulting from the pandemic and limited access to daily grocery shopping may have increased the probability of problematic eating habits [1, 2]. Furthermore, the COVID-19 pandemic might have limited the ability of patients with obesity to maintain a healthy lifestyle and to adhere to recommended ambulatory care plans. Accordingly, it seems to be reasonable to assume that the COVID-19 pandemic had a negative impact on the short-term results of bariatric surgeries.

Existing research on the pandemic’s effect on MBS outcomes is inconclusive. While some studies reported a negative effect of the COVID-19 pandemic on post-surgery weight loss [3–6], other studies found no such effect [7–13]. However, most of these studies have limitations, such as small sample sizes (below 210) [3, 4, 7, 9, 10, 12, 13] or study participants from only one study center [3–5, 7–13]. Available studies also primarily focus on weight loss outcomes (e.g., excess weight loss) and ignore other potential dimensions of MBS results (e.g., quality of life).

This study aims to assess the impact of the COVID-19 pandemic on 1-year outcomes of MBS in Germany. For that purpose, we analyzed a large dataset from a German national registry that includes bariatric surgery patients from 154 centers that report data to the DGAV (Deutsche Gesellschaft für Allgemein-und Viszeralchirurgie, German Society for general and visceral surgery). The primary outcomes were percentage total weight loss and quality of life 1-year post surgery. To our knowledge, we are the first to use such a comprehensive dataset to answer this research question and the first to extend this research question by the effect on quality of life 1-year post MBS.

## Methods

We conducted a retrospective analysis of a prospectively maintained dataset. Significant preliminary work for this study has been conducted in the context of a master`s thesis by Sophie Merzweiler at TU Munich. For the master thesis an a priori study protocol was created and uploaded to Open Science Framework (https://osf.io/r5gmq/) on 2023-07-03. Reporting followed STROBE guidelines (Supplementary Material 1).

### Data Source

Data from the prospective national German registry of the DGAV for obesity and metabolic surgery, StuDoQ|MBE, has been analyzed for this study. The Study, Documentation, and Quality Centre (Studien-, Dokumentations- und Qualitätszentrum, StuDoQ) of the DGAV receives clinical data from all certified, but only from individual (not all) non-certified centers for MBS across Germany [14], covering ~ 86% of all MBS between 2018 and 2022. Data collection is done routinely during the examinations at the bariatric clinic. If patients consent, their data will be forwarded to the registry [15]. Bariatric patients complete a self-administered questionnaire during the initial interview and at the post-surgery follow-up appointments (3-month and afterwards annually). The standardized patient questionnaires include e.g. sociodemographic questions, health questions and an adapted Bariatric Quality of Life Index (BQL) questionnaire [16, 17]. Furthermore, medical histories alongside different physical examinations are documented during the visits. After DGAV-StuDoQ approval, we received data for surgeries conducted between Mar 2018 and Dec 2021.

### Study Population and Exposure

Our exposure of interest was the COVID-19 pandemic and associated measures such as lockdowns, social distancing measures, etc. To analyze the effect of our exposure on outcomes, we compared two distinct groups of patients, a COVID-19 group and a No-COVID-19 group. Patients in the COVID-19 group had surgery between 2019-12-01 and 2020-02-29 and experienced the first (March 2020 - May 2020) and parts of the second (December 2020 - May 2021) lockdown in Germany during their first year post-surgery. Patients who underwent surgery in the same period one year earlier (2018-12-01 and 2019-02-28) constitute the No-COVID-19 group. Further information regarding COVID-19 measures in Germany and the rationale of the choice of the groups can be found in Supplementary Material 2.

Accordingly, our study population comprises all adult patients who underwent gastric bypass surgery (proximal and distal) or sleeve gastrectomy between 2018-12-01 and 2019-02-28 or 2019-12-01 and 2020-02-29 in one of the participating study centers of StuDoQ|MBE in Germany and who agreed that their data is documented in the STUDOQ database.

### Outcomes

We defined %TWL to be one of our primary outcomes. Preoperative weight and weight at 1-year follow-up are used to calculate %TWL.

Total weight loss (TWL) is defined as follows:

$$\:TWL=\:(preoperative\:weight\:-\:follow\:up\:weight)$$

Based on TWL, %TWL is calculated as:

$$\:\%TWL\hspace{0.17em}=\hspace{0.17em}100\:\times\:\:\frac{(preoperative\:weight\:-\:follow\:up\:weight)}{preoperative\:weight}$$

We defined BQL to be our second primary outcome. In the StuDoQ dataset, patients` responses to the 13 items of the second part of the validated BQL questionnaire were available [16]. The patients rate each of these 13 items using a Likert scale from 1 to 5. The overall value of the BQL was measured by calculating the mean of the 13-item scale:

$$\:BQL=\:\frac{1}{13}\sum\:_{i=1}^{13}{BQL\:item}_{i}$$

BQL ranges from 1 to 5, with 1 corresponding to the lowest and 5 to the highest possible quality of life score. The corresponding questionnaire and more information regarding BQL and the coding of the items can be found in Supplementary Material 3.

As secondary outcomes, we analyzed participation in the 3-month and 1-year follow-up examination, T2D and hypertension at 1-year follow-up examination. Further information on secondary outcomes can be found in Supplementary Material 4.

### Statistical Analysis

Analyses were performed in R (v4.3.3). The complete analysis code is documented in a supplemantary RMarkdown document (Supplementary Material 2). Baseline characteristics (age, sex, BMI, education, employment, type of surgical procedure, T2D, hypertension, dyslipidemia and obstructive sleep apnea (OSA)) were used for group comparisons and regression adjustment. Group differences in %TWL and BQL were tested using Wilcoxon tests; secondary outcomes via χ² tests. Multivariable linear and logistic regression models estimated the effect of COVID-19, adjusting for confounders. Effect estimates, odds ratios, 95% CIs, and p-values are reported. A p-value of < 0.05 was considered statistically significant.

Our dataset contained a considerable amount of missing data. Especially for our BQL outcome and for some of the baseline characteristics more than 50% of the data was missing. However, when comparing the share of missing information between our two study groups, no structural differences became apparent. Table S1 in Supplementary Material 6 summarizes our analysis of missing values. We decided to conduct a complete case analysis. A flowchart for sample construction is available in Supplementary Material 6. Further details on how we dealt with missing information is available in the supplementary RMarkdown document.

## Results

### Descriptive Analysis

A total of 5,859 patients met the eligibility criteria, with 3,195 in the COVID-19 group and 2,664 in the No-COVID-19 group. Table 1 summarizes baseline characteristics for the overall sample and by group.

**Table 1:** Baseline characteristics of the study population

	Overall (*n* = 5859)	No-COVID-19 (*n* = 2664)	COVID-19 (*n* = 3159)
**Sex** (= female)	70.7%	71.1%	70.4%
**Age** in years (Mean)	43.9	44.1	43.7
**Weight** in kg (Mean)	140.3	141.0	139.7
**BMI** (Mean)	48.5	48.6	48.4
**Graduation**			
High	15.3%	14.6%	15.8%
Intermediate	33.5%	34.3%	32.9%
Low	26.0%	26.8%	25.4%
Without	4.1%	4.4%	3.9%
Unknown	21.1%	19.9%	22.0%
**Employment**			
Employed	55.6%	54.0%	56.9%
Housewife/Househusband	7.6%	8.0%	7.3%
Retired	7.6%	8.0%	7.2%
Not employed	16.1%	16.9%	15.5%
Unknown	13.1%	13.1%	13.1%
**Type of surgical procedure**			
Bypass	37.5%	40.3%	35.2%
Sleeve	62.5%	59.7%	64.8%
**T2D** (present)	25.6%	26.0%	25.3%
**Hypertension** (present)	57.5%	58.3%	56.7%
**Dyslipidemia** (present)	18.8%	18.4%	19.2%
**OSA** (present)	34.1%	34.4%	33.8%

Descriptive overview of baseline characteristics for the overall study population and subdivided by study groups (No-COVID-19, COVID-19). The table shows relative frequencies (%) for categorical characteristics and means for metric characteristics. The total group size is indicated by n (sample size). Due to missing data, the numbers presented in the table are not always based on the total group size but are based on those observations for which the respective information is not missing. More information on missing data is available in Supplementary Material 5. Graduation refers to graduation levels in Germany: “Low”= Hauptschule; “Intermediate”= Mittlere Reife, “High”= Hochschulreife (A-levels)). Other abbreviations: Bypass= gastric bypass, Sleeve= sleeve gastrectomy, T2D= Type 2 Diabetes, OSA= obstructive sleep apnea

Patients were aged 18–81 years (mean: 43.9); 70.7% were female. Sleeve gastrectomy was the most common procedure (62.5%). Mean preoperative weight was 140.3 kg, mean BMI 48.5 kg/m². Most patients had intermediate-level education (33.5%) and were employed (55.6%). Associated medical problems included T2D (25.6%), hypertension (57.5%), dyslipidemia (18.8%), and OSA (34.1%). No structural differences in baseline characteristics were observed between study groups.

Table 2 compares primary and secondary outcomes. Mean %TWL was similar across groups (31.1% vs. 31.3%; *p* = 0.530). BQL was slightly lower in the COVID-19 group (4.01 vs. 4.12; Δ = − 0.11; *p* = 0.019).

**Table 2:** Descriptive overview of outcomes for No-COVID-19 and COVID-19 group at the 1 year follow-up

	*N*	No-COVID-19	COVID-19	Group Difference	*p*-value
**Primary Outcomes**					
%TWL	3901	31.1% (10.1)	31.3% (10.1)	0.2%	0.530
BQL	1218	4.12 (0.65)	4.01 (0.73)	−0.11	0.006
***Secondary Outcomes***					
3-month fu	5838	82.7%	75.3%	−7.4%	< 0.001
1-year fu	5804	71.3%	63.7%	−7.7%	< 0.001
T2D	3892	18.5%	17.8%	−0.7%	0.611
Hypertension	3892	41.6%	38.6%	−3.0%	0.057

Descriptive overview of the primary and secondary outcomes for the No-COVID-19 and COVID-19 study groups. The sample size on which the respective outcome comparison is based on is indicated in the N column and in Supplementary Material 6. The table shows the mean, standard deviation (SD), group difference (COVID-19 group - No-COVID-19 group), and the p-value for the Wilcoxon test for the primary outcomes. BQL was measured with the BQL questionnaire. A score of 5 correlates to a high BQL, while a score of 1 indicates a low BQL. For the secondary outcomes, the table shows relative frequencies, the difference in relative frequency between the No-COVID-19 and COVID-19 study groups, and the p-value of Pearson’s chi-squared test. Other abbreviations: %TWL= Percentage total weight loss; BQL= Bariatric Quality of Life; fu= follow-up examination; T2D= Type 2 Diabetes

Follow-up participation was significantly reduced in the COVID-19 group: 3-month (75.3% vs. 82.7%; *p* < 0.001) and 1-year (63.7% vs. 71.3%; *p* < 0.001). T2D prevalence at 1-year was ~ 18% in both groups; hypertension was slightly lower in the COVID-19 group (38.6% vs. 41.6%), but differences were not statistically significant.

### Regression Analysis

Table 3 summarizes regression results with a focus on the effect of the COVID-19 pandemic. The estimated coefficients for the included covariates are available in the R-Markdown mentioned above (XYZ). No significant association was found between COVID-19 and %TWL (*p* = 0.353). The COVID-19 pandemic was associated with a significant 0.14 decrease in BQL (*p* < 0.001).

**Table 3:** Regression model results

	*N*	Model	COVID-19 effect	95%-CI	*p*-value
**Primary Outcomes**			**β**		
%TWL	3901	Linear	0.29	[−0.32, 0.89]	0.353
BQL	1218	Linear	−0.14	[−0.21, −0.07]	< 0.001
***Secondary Outcomes***			**OR**		
Participation 3-month fu	5816	Logistic	0.62	[0.54, 0.70]	< 0.001
Participation 1-year fu	5782	Logistic	0.69	[0.62, 0.77]	< 0.001
T2D	3892	Logistic	1.01	[0.84, 1.20]	0.945
Hypertension	3892	Logistic	0.89	[0.77, 1.03]	0.126

The table displays the results of the multivariable regression analyses described in the methods section. N specifies the sample size on which the relevant model is dependent. The model type column shows whether a linear or logistic regression was carried out. The β parameter estimate represents the COVID-19 effect for the primary outcomes, while the Odds Ratio (OR) represents the COVID-19 effect for the secondary outcomes, along with the 95%-confidence interval (95%-CI) and p-value. The complete regression results including the estimated beta coefficients for the included covariables are available in the RMarkdown document that is available on the Open Science Framework (osf.io/r5gmq/). Other abbreviations: %TWL= Percentage total weight loss; QoL= Quality of life; fu= follow-up examination; T2D= Type 2 Diabetes

COVID-19 was associated with reduced odds of follow-up participation: 3-month (OR = 0.62; *p* < 0.001) and 1-year (OR = 0.69; *p* < 0.001). No significant associations were found for T2D (*p* = 0.945) and hypertension (*p* = 0.126).

## Discussion

The study provides evidence that patients that continued to participate in their follow-up visits despite the COVID-19 pandemic had a similar %TWL to patients prior the pandemic. However, the first two pandemic waves led to a small but statistically significant reduction in BQL. Regarding secondary outcomes, the pandemic significantly decreased participation rates in both 3-month and 1-year follow-ups. No significant associations were found for T2D or hypertension prevalence at 1-year post-surgery.

That we did not find an effect of the COVID-19 pandemic on %TWL, is contrary to our hypothesis. However, one possible explanation of this null finding is that MBS leads to such fundamental physiological changes [18] that external factors like a pandemic have little to no influence during the first year post-surgery.

In line with our hypothesis, COVID-19 had a measurable impact on BQL. Lockdown measures limited social interaction and access to group activities, potentially leading to feelings of exclusion and reduced quality of life among MBS patients. Additionally, early reports of increased COVID-19 risk for obese individuals may have heightened fear and anxiety in this population [19, 20]. These factors could have contributed to emotional distress, including depression, dissatisfaction with weight, and increased vulnerability to loneliness and anxiety [21]. However, the observed effect on BQL is small and probably not clinically meaningful. Furthermore, it is quite likely that this small effect diminished quite soon after the pandemic and the associated restrictions have ended. The unexpectedly small effect on BQL may be partly explained by our findings for the %TWL outcome. During the first postoperative year, quality of life in MBS patients is likely to be strongly driven by the physiological effects of surgery. Consequently, the absence of an effect of the pandemic on %TWL may, at least in part, explain the limited effect observed for BQL.

As expected, we observed significant differences between the COVID-19 and No-COVID-19 group for participation in follow-up examinations. During the first and second lockdown, patients might have had limited access to follow-up examinations at bariatric clinics due to COVID-19 restrictions. Additionally, patients may have missed a follow-up examination due to the fear of a COVID-19 infection or could not attend due to a COVID-19 infection. These findings highlight the importance of maintaining continuity of care during public health crises. In future pandemics, healthcare systems should ensure that follow-up care for MBS patients remains accessible, for example through telemedicine or flexible scheduling. Clear communication about safety measures and alternative follow-up options may help reduce anxiety and improve adherence.

Contrary to what we expected, we did not observe a significant effect of the COVID-19 pandemic on our secondary outcomes T2D and hypertension. This could be due to the fundamental physiological changes caused by bariatric surgery mentioned above [18].

### Comparison with Previous Findings

When comparing our results to existing studies, it is important to note that COVID-19 measures and study populations varied across countries. Additionally, some studies only included patients who had either sleeve [4] or bypass surgery [3].

Our finding that there was no significant effect of the pandemic on %TWL 1-year post surgery is consistent with previous research that also included patients in the COVID-19 exposed group who underwent surgery up to six months prior to the pandemic (October 2019 to March 2020) [3, 8]. It is also consistent with findings from studies that consider a follow-up of 2 [13] or 3 years [12]. Contrary to the findings of this study, El Moussaoui et al. [4] found a significant difference for 1-year %TWL. However, their COVID-group included patients who underwent surgery between June and October 2019, which means that there was a longer break between surgery and the outbreak of the pandemic.

The observed reduction in BQL is consistent with Sisto et al. [22], who found increased psychological stress among bariatric patients during the pandemic, leading to a deterioration in quality of life.

Contrary to the findings of Barranquero et al. [3], we found differences in the participation in 3-month and 1-year follow-up examinations between the groups. However, a similar pattern to ours that the non-attendance rate significantly varied between the COVID-19 and No-COVID group was reported by Vitiello et al. [5].

Consistent with prior studies [3–6, 11–13], we found no significant differences in T2D and hypertension rates.

### Strengths and Limitations of the Study

To our knowledge, this is the most extensive study to date examining the impact of COVID-19 on short-term results of MBS. With over 80% of bariatric patients included in the registry, the study is nationally representative for Germany. We are also the first to assess the pandemic’s effect on quality of life in this patient group.

Due to the nature of the exposure, we used a non-randomized, observational design with a historical control group. While this design carries a risk of bias from unobserved confounders, patients in the COVID-19 group were unaware of the upcoming pandemic at the time of surgery, which helps mitigate this issue. Including a broad set of observed covariates (e.g., education, surgery type) also had minimal impact on effect estimates, suggesting limited unobserved confounder bias.

Differences in loss-to-follow-up rates present another potential source of bias. The COVID-19 group had a higher loss to follow-up rate at 1 year (36.7% vs. 28.7%). If patients lost to follow-up differed systematically from those who attended the 1-year follow-up in terms of bariatric surgery outcomes, this discrepancy could have biased our results. However, an analysis of baseline characteristics revealed no systematic differences between patients lost to follow-up and those who completed the 1-year follow-up. Additionally, findings from Krietenstein et al. [23] indicate that %TWL and improvements in associated medical problems at 1-year follow-up were comparable for patients who underwent bariatric surgery between 2008 and 2017 in Germany and were lost to follow-up. Nevertheless, the 8% increase in loss to follow-up during the Covid-19 pandemic might reflect a different patient population than those typically lost to follow-up. It remains unclear whether these patients would have experienced better, worse, or similar outcomes compared to regular lost-to-follow-up patients, likely representing a heterogeneous mix. Consequently, while the differing loss-to-follow-up rates may introduce bias, the direction of this bias is uncertain. Overall, we consider the respective risk of bias to be rather low in our study.

Another limitation concerns the interpretation of our BQL results: We cannot compare the impact of the COVID-19 pandemic on the quality of life of MBS patients with its impact on the general population. It is evident that the pandemic also affected the general population’s quality of life, and it would have been valuable to assess whether MBS patients were more, less, or equally affected. Unfortunately, due to the unavailability of suitable data, such a comparison was not possible.

An additional limitation is that we only look at a follow-up period of one year.

## Conclusion

This study is, to our knowledge, the largest to examine the impact of the COVID-19 pandemic on bariatric surgery outcomes 1-year post-surgery. While COVID-19 had no significant effect on %TWL, it was associated with a small but statistically significant reduction in quality of life and a substantial decrease in follow-up participation.

These findings offer important insights into how pandemic-related disruptions affect short-term outcomes of MBS and highlight the need to ensure continuity of care during future public health crises.

## Supplementary Information

Below is the link to the electronic supplementary material.

Supplementary Material 1 (DOCX 696 KB)

Supplementary Material 2 (HTML 1.08 MB)

## Data Availability

All relevant data is available within the manuscript and in the submitted figures and tables. Further inquiries can be directed at the corresponding author.
