# Supplementary material for: The Impact of the COVID-19 Pandemic on Weight Loss and Quality of Life One Year Post Metabolic Bariatric Surgery
Source: Obes Surg. 2026 Mar 9;36(4):1503–9. doi: 10.1007/s11695-026-08515-6 (PMC13083447; doi:10.1007/s11695-026-08515-6)
Supplement: Supplementary file 1 — Supplementary Material 1 (DOCX 696 KB) [file 11695_2026_8515_MOESM1_ESM.docx]

**Impact of the COVID-19 pandemic on weight loss and quality of life one year post bariatric surgery**

**Supplementary material**

Supplementary material 1: STROBE statement - checklist

Supplementary material 2: Rational for the choice of study groups

Supplementary material 3: Information about Bariatric Quality of Life Index

Supplementary material 4: Information regarding secondary outcomes

Supplementary material 5: Overview of missing values

Supplementary material 6: Flowchart of sample construction

Supplementary Material 1: STROBE statement - checklist

STROBE Statement—checklist of items that should be included in reports of observational studies

|  | **Item No** | **Recommendation** | **Page  No** |
| --- | --- | --- | --- |
| **Title and abstract** | 1 | (*a*) Indicate the study’s design with a commonly used term in the title or the abstract | 2 |
|  |  | (*b*) Provide in the abstract an informative and balanced summary of what was done and what was found | 2 |
| **Introduction** | | | |
| Background/rationale | 2 | Explain the scientific background and rationale for the investigation being reported | 4 |
| Objectives | 3 | State specific objectives, including any prespecified hypotheses | 4 |
| **Methods** | | | |
| Study design | 4 | Present key elements of study design early in the paper | 5 |
| Setting | 5 | Describe the setting, locations, and relevant dates, including periods of recruitment, exposure, follow-up, and data collection | 5-6 |
| Participants | 6 | (*a*) *Cohort study*—Give the eligibility criteria, and the sources and methods of selection of participants. Describe methods of follow-up  *Case-control study*—Give the eligibility criteria, and the sources and methods of case ascertainment and control selection. Give the rationale for the choice of cases and controls  *Cross-sectional study*—Give the eligibility criteria, and the sources and methods of selection of participants | 5-6 |
|  |  | (*b*) *Cohort study*—For matched studies, give matching criteria and number of exposed and unexposed  *Case-control study*—For matched studies, give matching criteria and the number of controls per case |  |
| Variables | 7 | Clearly define all outcomes, exposures, predictors, potential confounders, and effect modifiers. Give diagnostic criteria, if applicable | 6-7 |
| Data sources/ measurement | 8* | For each variable of interest, give sources of data and details of methods of assessment (measurement). Describe comparability of assessment methods if there is more than one group | 6-7 |
| Bias | 9 | Describe any efforts to address potential sources of bias | 7 |
| Study size | 10 | Explain how the study size was arrived at | X |
| Quantitative variables | 11 | Explain how quantitative variables were handled in the analyses. If applicable, describe which groupings were chosen and why | 6-7  SM3, SM4, R Code |
| Statistical methods | 12 | (*a*) Describe all statistical methods, including those used to control for confounding | 7 |
|  |  | (*b*) Describe any methods used to examine subgroups and interactions | 7 |
|  |  | (*c*) Explain how missing data were addressed | 7,  R Code |
|  |  | (*d*) *Cohort study*—If applicable, explain how loss to follow-up was addressed  *Case-control study*—If applicable, explain how matching of cases and controls was addressed  *Cross-sectional study*—If applicable, describe analytical methods taking account of sampling strategy |  |
|  |  | (*e*) Describe any sensitivity analyses |  |

| **Results** | | | |
| --- | --- | --- | --- |
| Participants | 13* | (a) Report numbers of individuals at each stage of study—eg numbers potentially eligible, examined for eligibility, confirmed eligible, included in the study, completing follow-up, and analysed | 7-8 |
|  |  | (b) Give reasons for non-participation at each stage | 7-8 |
|  |  | (c) Consider use of a flow diagram | SM6 |
| Descriptive data | 14* | (a) Give characteristics of study participants (eg demographic, clinical, social) and information on exposures and potential confounders | 7-8 |
|  |  | (b) Indicate number of participants with missing data for each variable of interest | SM5 |
|  |  | (c) *Cohort study*—Summarise follow-up time (eg, average and total amount) |  |
| Outcome data | 15* | *Cohort study*—Report numbers of outcome events or summary measures over time | *8* |
|  |  | *Case-control study—*Report numbers in each exposure category, or summary measures of exposure |  |
|  |  | *Cross-sectional study—*Report numbers of outcome events or summary measures |  |
| Main results | 16 | (*a*) Give unadjusted estimates and, if applicable, confounder-adjusted estimates and their precision (eg, 95% confidence interval). Make clear which confounders were adjusted for and why they were included | 8-9 |
|  |  | (*b*) Report category boundaries when continuous variables were categorized |  |
|  |  | (*c*) If relevant, consider translating estimates of relative risk into absolute risk for a meaningful time period |  |
| Other analyses | 17 | Report other analyses done—eg analyses of subgroups and interactions, and sensitivity analyses |  |
| **Discussion** | | | |
| Key results | 18 | Summarise key results with reference to study objectives | 9-10 |
| Limitations | 19 | Discuss limitations of the study, taking into account sources of potential bias or imprecision. Discuss both direction and magnitude of any potential bias | 11-12 |
| Interpretation | 20 | Give a cautious overall interpretation of results considering objectives, limitations, multiplicity of analyses, results from similar studies, and other relevant evidence | 9-11 |
| Generalisability | 21 | Discuss the generalisability (external validity) of the study results | 11 |
| **Other information** | | | |
| Funding | 22 | Give the source of funding and the role of the funders for the present study and, if applicable, for the original study on which the present article is based | 14 |

*Give information separately for cases and controls in case-control studies and, if applicable, for exposed and unexposed groups in cohort and cross-sectional studies.

von Elm, E., Altman, D. G., Egger, M., Pocock, S. J., Gøtzsche, P. C., & Vandenbroucke, J. P. (2007). The Strengthening the Reporting of Observational Studies in Epidemiology (STROBE) statement: guidelines for reporting observational studies. *Lancet*, *370*(9596), 1453-1457. <https://doi.org/10.1016/s0140-6736(07)61602-x>

Supplementary Material 2: Rational for the choice of study groups

The **COVID-19 group** was chosen because bariatric surgery took place shortly before the first national COVID-19 lockdown from March to May 2020 in Germany. At the time of surgery, it was not foreseeable that life would change drastically in the near future. These patients experienced the first and parts of the second lockdown and did not experience a lockdown before in Germany. In March 2020, the World Health Organization declared the COVID-19 (Corona Virus Disease) outbreak a pandemic (World Health Organization 2020). Germany was also affected by the pandemic in spring 2020. With the goal of reducing contacts and thus stopping the dynamic spread of COVID-19, numerous "social distancing" measures were implemented. According to the Infection Protection Act, the government can impose limitations or temporarily suspend fundamental liberties of the population (German Federal Government 2001).

The first significant lockdown in Germany started on 22 March 2020 and lasted until the beginning of May 2020 (Presse- und Informationsamt der Bundesregierung 2020) (Presse- und Informationsamt der Bundesregierung 2020). The following measures were for example initially mandated: Avoiding contact, staying at home, keeping a large distance from other people, restaurants, and services were closed (e.g. the closure of all sporting venues) (Presse- und Informationsamt der Bundesregierung 2020, Michelini, Bortoletto et al. 2021). During the lockdown, individuals were allowed to engage in some outdoor sports and physical activities alone but not with others (Presse- und Informationsamt der Bundesregierung 2020, Michelini, Bortoletto et al. 2021). After the first strict lockdown, the first easing was decided. For instance, non-contact individual sports with distance (e.g. tennis, athletics, golf, sailing, and horseback riding were allowed in Bavaria from 11 May onwards (Bayerisches Staatsministerium des Innern 2020). Further easements began at the end of May or the start of June depending on the region of Germany. In some federal states, fitness studios were allowed to reopen under strict conditions and contact sports were possible outside. For instance, gyms and sports facilities were allowed to open again in Baden-Wurttemberg for training and exercise purposes on May 11. Regulations such as a minimum distance of 1.5 m, maximum number of people, etc., have been introduced (Ministerium für Kultus - Jugend und Sport - Baden-Württemberg 2020).

The second lockdown in Germany took place from December 2020 till May 2021. Before the second lockdown, a lockdown-light was mandated. The aim of the lockdown light was to reduce contacts to an absolute minimum. Aside from individual sports played alone, in couples, or with members of one's own home, all institutions and facilities associated with recreational activities were shut down on the November 2, 2020. This included recreational and amateur sports played in all public and private sporting facilities (Presse- und Informationsamt der Bundesregierung 2020). Due to the once again exponentially rising infection figures and the increasingly highly critical situations in the hospitals, federal and state governments agreed on extensive measures starting December 16, 2020 to prevent further escalations of infections (Presse- und Informationsamt der Bundesregierung 2020, Staatsministerium Baden-Württemberg 2020). Initially, the measures were to apply until January 10, 2021, but have been repeatedly extended (Presse- und Informationsamt der Bundesregierung 2020, Presse- und Informationsamt der Bundesregierung 2021, Presse- und Informationsamt der Bundesregierung 2021, Presse- und Informationsamt der Bundesregierung 2022). The strict measures were lifted at the end of May 2021 (Bundesministerium für Gesundheit 2023).

The **No-COVID-19 group** consists of bariatric patients who underwent surgery in the same period of the previous year and therefore did not experience the COVID-19 lockdown and regulations in Germany. Our assumption is that individuals from the COVID-19 group are very similar to the individuals included in the **No-COVID-19 group**. We are confident that this assumption holds since individuals in both groups were operated in the same months and before the pandemic hit Germany.

Supplementary Material 3: Information about Bariatric Quality of Life Index

**STUDOQ BQL (German Version)**


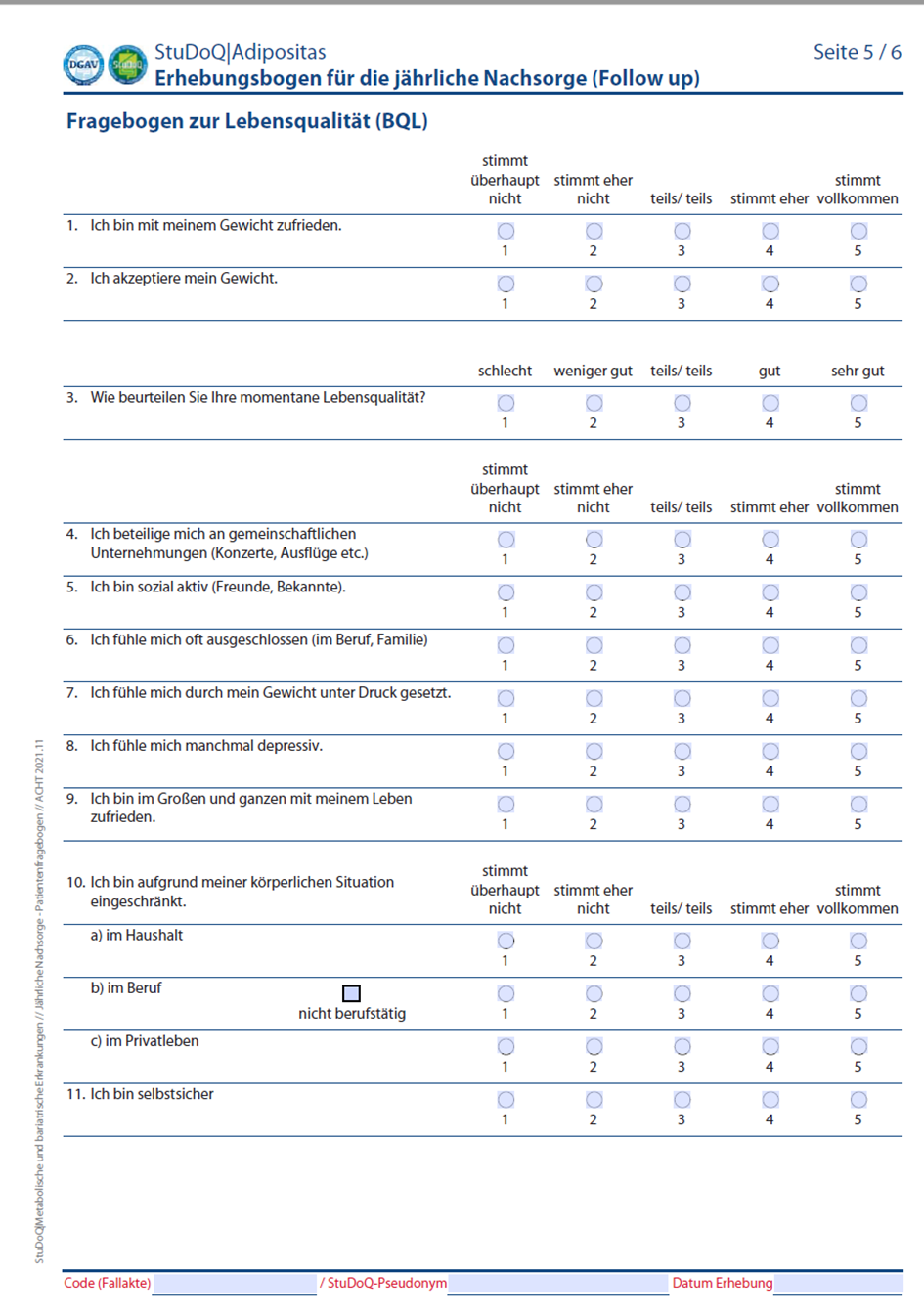


**English Version of STUDOQ BQL**

|  | **Absolutely wrong** | **wrong** | **half/half** | **true** | **Absolutely right** |
| --- | --- | --- | --- | --- | --- |
| **1. I like my weight.** | o1 | o2 | o3 | o4 | o5 |
| **2. I can accept my weight.** | o1 | o2 | o3 | o4 | o5 |

|  | **bad** | **Less good** | **half/half** | **good** | **Very good** |
| --- | --- | --- | --- | --- | --- |
| **3. How is your actual quality of life?** | o1 | o2 | o3 | o4 | o5 |

|  | **Absolutely wrong** | **wrong** | **half/half** | **true** | **Absolutely right** |
| --- | --- | --- | --- | --- | --- |
| **4. I am participating in social activities (theatre, etc.).** | o1 | o2 | o3 | o4 | o5 |
| **5.I often meet friends or family.** | o1 | o2 | o3 | o4 | o5 |
| **6. I feel excluded from social life.** | o1 | o2 | o3 | o4 | o5 |
| **7. I feel under pressure because of my weight.** | o1 | o2 | o3 | o4 | o5 |
| **8. Sometimes, I feel depressed.** | o1 | o2 | o3 | o4 | o5 |
| **9. All in all, I feel satisfied in my life.** | o1 | o2 | o3 | o4 | o5 |

| **10. I feel restricted because of my weight.** | | **Absolutely wrong** | **wrong** | **half/half** | **true** | **Absolutely right** |
| --- | --- | --- | --- | --- | --- | --- |
| **a) at home** | | o1 | o2 | o3 | o4 | o5 |
| **b) at work** | **o not employed** | o1 | o2 | o3 | o4 | o5 |
| **c) privately** | | o1 | o2 | o3 | o4 | o5 |
| **11. I feel self-confident.** | | o1 | o2 | o3 | o4 | o5 |

In our study, quality of life was measured by means of the Bariatric Quality of Life Index (BQL) (Weiner, Sauerland et al. 2005). The BQL covers the following domains: psychological well-being, social functioning, physical functioning, problems and symptoms related to obesity surgery, and obesity-related co-morbidity. Weiner, Sauerland et al. (2009) claim that the BQL is a valid tool. The BQL can be divided into a 16-item non-QoL section (Yes/No answers) and a 14-item QoL section (Likert-scale). In the StuDoQ|MBE questionnaire, only the second part (QoL) of the BQL questionnaire was used. However, Item 4 (“I exercise regularly.”) from the 14-item section (QoL) of the original questionnaire was removed due to findings of a validation study from 2009 (Weiner, Sauerland et al. 2009). The results of a factor analysis showed that after removing item 4, Cronbach's α increased to 0.898 . Since Cronbach's alpha is 0.93 for 12 months after surgery, contingency of the BQL/ internal consistency is good. Pearson test r = 0.9 indicates strong retest reliability. In the validation study the 16-item scale (NQoL) data and the 14-item scale (QoL) data were treated completely separately (Weiner, Sauerland et al. 2009).

The overall value of the BQL was determined by calculating the mean of the Likert-scale (1-5) of the 13-item likert scale. Therefore the answers of questions (“ absolutely wrong”, “wrong“, “half/half“, “true“, “absolutely right”) or( “bad”, “less good”, “half/half”, “good”, “very good”) were recoded into likert scale level 1-5. For questions 6, 7, 8, 10a, 10b, 10c the Likert scale was inverted, because these questions were formulated in the opposite way. For these items “absolutely wrong” is good (5) and “absolutely right” (1) is bad.

Supplementary Material 4: Information regarding secondary outcomes

**Participation in follow-up examination 3-months and 1-year post surgery:** The data set contained information on whether or not a patient participated in the follow-up. After exploring the data, we decided to define our two secondary outcome variables in the following way :

- **3-month appointment:** If weight was available for the 3-month appointment and attendance at the 3-month appointment has the category "clinic change" the response for attendance at the 3-month appointment was recoded to "yes" because the weight was measured and transmitted to the registry. If the weight was not available, the observations were recoded to be NA. If weight was available for the 3-month appointment, participation 3-month appointment was recoded from no to yes. The category NA for participation in follow-up was recoded to "no" because no information was submitted to the registry. Therefore, it was assumed that these follow-up examinations did not take place.
- **1-year follow-up appointment:** The category "yes, interim care" was set to "yes" as data for 1-year follow-up appointment was present. The category NA for participation in follow-up is recoded to "no" because no information was submitted to the registry. Therefore, it is assumed that these follow-up examinations did not take place. If weight was available for the 1-year appointment and attendance at the 1-year appointment has the category "clinic change," the response for attendance at the 1-year appointment was recoded to "yes" because the weight was measured and transmitted to the registry. If weight was not available, the observations were later recoded to NA. If weight was available for the one-year appointment, participation one-year appointment was recoded from no to yes.

**Type 2 Diabetes (T2D) and hypertension:** In addition, we investigated whether T2D and hypertension occurred more or less frequently during the COVID-19 pandemic at the time of the 1-year follow-up examination. In the “survey for the yearly check-up following your surgery” the patients were asked for example whether they have T2D and hypertension or not. The secondary outcomes T2D and hypertension were coded as binary outcomes (yes/ no). The yes category included the following answers: Yes, same as before surgery; Yes, better than before surgery; Yes, worse than before surgery; Yes, new occurrence.

**Questionnaire: Questions about your health:**

**Do you have the following illnesses (please tick)?**

|  | *No* | *Yes, same as before surgery* | *Yes, better than before surgery* | *Yes, worse than before surgery* | *Yes, new occurrence* |
| --- | --- | --- | --- | --- | --- |
| Type 1 Diabetes |  |  |  |  |  |
| Type 2 Diabetes |  |  |  |  |  |
| High blood pressure |  |  |  |  |  |
| Sleep apnea (stopped breathing while sleeping) |  |  |  |  |  |
| Lipid metabolism disorder |  |  |  |  |  |
| Increased uric acid / gout |  |  |  |  |  |
| Polycystic Ovary Syndrome (PCOS) |  |  |  |  |  |
| Joint diseases/pain  *-> Which?* |  |  |  |  |  |
| Do you suffer from depression? |  |  |  |  |  |
| Do you suffer from urinary incontinence? |  |  |  |  |  |
| Do you suffer from heartburn? |  |  |  |  |  |

*German Questionnaire*


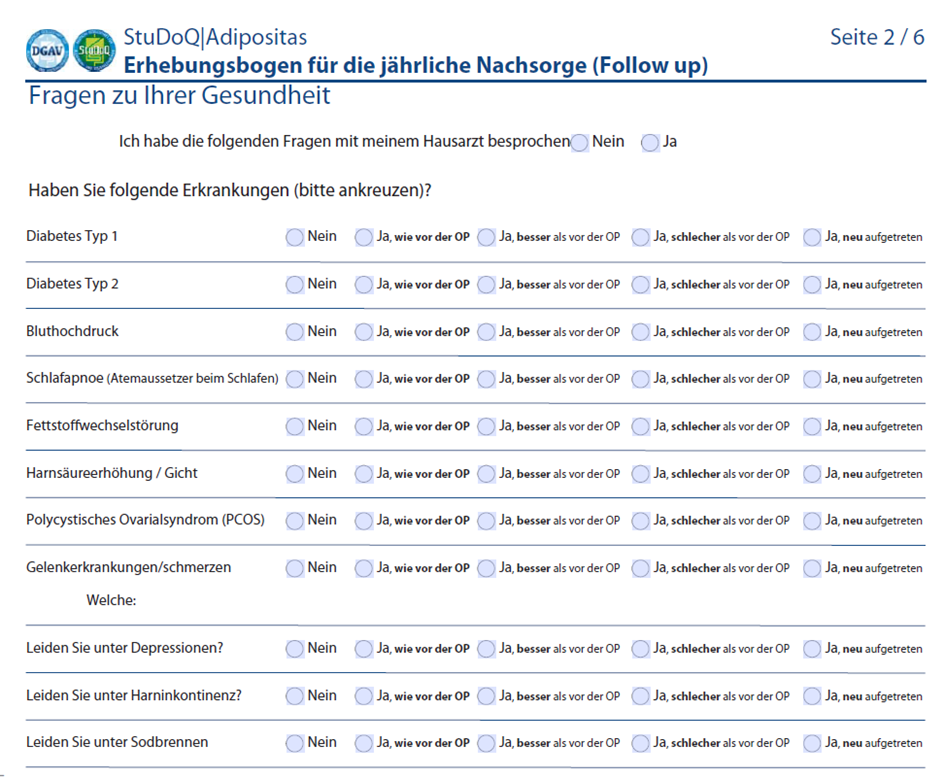


Supplementary Material 5: Overview of Missing Values

*Table S1: Overview of Missing Values*

| **Variable** | **Overall** | **No-COVID-19 group** | **COVID-19 group** |
| --- | --- | --- | --- |
| Sex | 0.03% | 0.04% | 0.03% |
| Age | 0.00% | 0.00% | 0.00% |
| Graduation | 24.63% | 25.64% | 23.79% |
| Employment | 24.54% | 25.60% | 23.66% |
| Weight | 0.38% | 0.83% | 0.00% |
| BMI | 0.38% | 0.83% | 0.00% |
| Type of surgical procedure | 0.00% | 0.00% | 0.00% |
| Diabetes type 2 at baseline | 59.53% | 56.64% | 61.94% |
| Hypertension at baseline | 59.53% | 56.64% | 61.94% |
| Dyslipidemia at baseline | 59.53% | 56.64% | 61.94% |
| Sleep_apnoea at baseline | 59.53% | 56.64% | 61.94% |
| Number of comorbidities at baseline | 59.53% | 56.64% | 61.94% |
| Participation in 3-month follow-up | 0.36% | 0.15% | 0.53% |
| Participation in 1-year follow-up | 0.94% | 0.53% | 1.28% |
| % TWL | 33.42% | 28.98% | 37.12% |
| BQL | 79.21% | 78.04% | 80.19% |
| Diabetes type 2 at follow-up | 33.57% | 29.35% | 37.09% |
| Hypertension at follow-up | 33.57% | 29.35% | 37.09% |
|  |  |  |  |

**Notes:** Descriptive overview of missing values for overall dataset and subdivided by subgroups. Table S1 shows relative frequencies (%) of missing values for each variable.

Supplementary Material 6: Flowchart of sample construction

*Figure S1: Sample Construction of Study Groups*


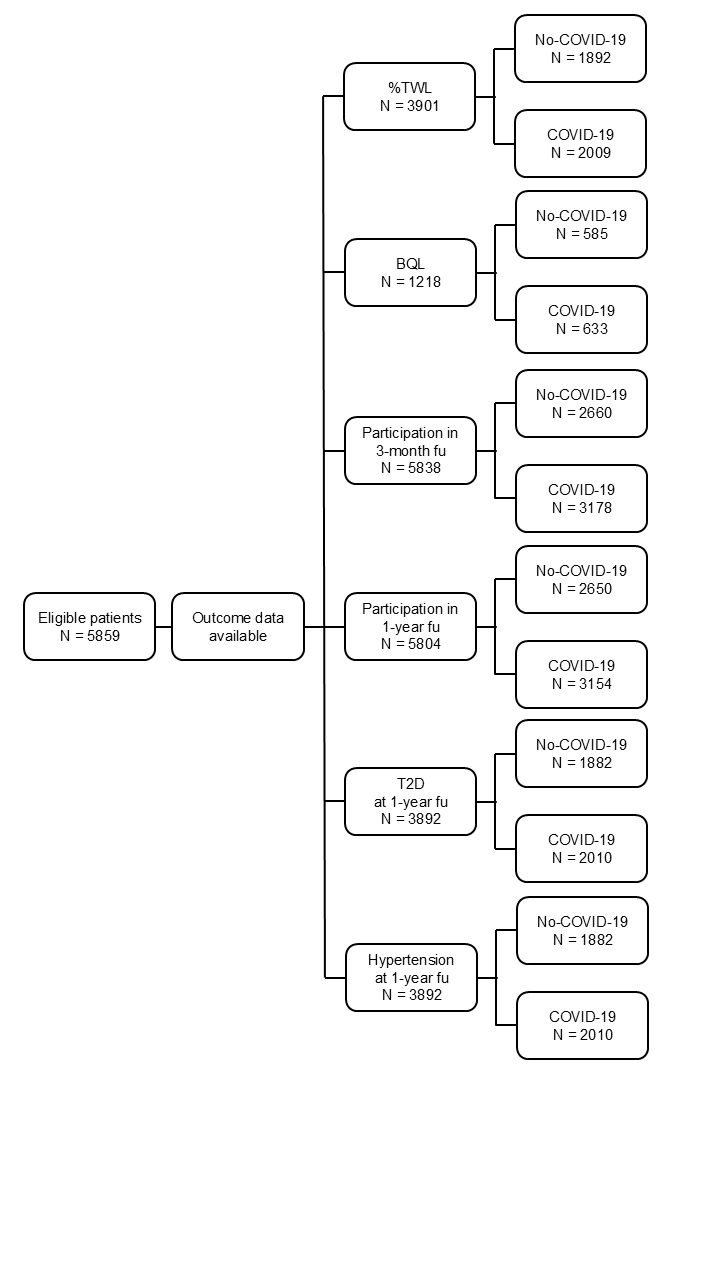


**Notes:** Figure S1 shows the sample sizes for the different outcomes and study groups.

**References**

Bayerisches Staatsministerium des Innern, f. S. u. I. (2020) Schrittweise Erleichterungen bei den Corona-Maßnahmen.

Bundesministerium für Gesundheit (2023) Coronavirus-Pandemie: Was geschah wann? Chronik Coronavirus

German Federal Government (2001). Infektionsschutzgesetz. **2126-13**.

Michelini, E., et al. (2021). "Outdoor Physical Activity During the First Wave of the COVID-19 Pandemic. A Comparative Analysis of Government Restrictions in Italy, France, and Germany." Front Public Health **9**: 615745.

Ministerium für Kultus - Jugend und Sport - Baden-Württemberg (2020) Verordnung des Kultusministeriums und des Sozialministeriums über Sportstätten (Corona-Verordnung Sportstätten – CoronaVO Sportstätten).

Presse- und Informationsamt der Bundesregierung (2020) 6. Mai 2020: Regeln zum Corona-Virus. Corona-Virus

Presse- und Informationsamt der Bundesregierung (2020) Besprechung der Bundeskanzlerin mit den Regierungschefinnen und Regierungschefs der Länder vom 22.03.2020. Coronavirus in Deutschland

Presse- und Informationsamt der Bundesregierung (2020) Corona Eindämmung: Diese Regeln gelten jetzt. Coronavirus in Deutschland

Presse- und Informationsamt der Bundesregierung (2020) "Wir sind zum Handeln gezwungen". Coronavirus in Deutschland

Presse- und Informationsamt der Bundesregierung (2021) Das regelt die bundeseinheitliche Notbremse. Coronavirus in Deutschland - Infektionsschutzgesetz

Presse- und Informationsamt der Bundesregierung (2021) Neue Corona-Maßnahmen gelten bundesweit. Bund-Länder-Beschluss vom 5.1.

Presse- und Informationsamt der Bundesregierung (2022, 21.03.2022). "Corona-Regelungen: Basis-Schutz und Hotspot-Maßnahmen." Coronavirus in Deutschland - Infektionsschutzgesetz. from <https://www.bundesregierung.de/breg-de/themen/coronavirus/corona-regeln-und-einschrankungen-1734724>.

Staatsministerium Baden-Württemberg (2020) Bund und Länder einigen sich auf Lockdown ab 16. Dezember. CORONAVIRUS

Weiner, S., et al. (2005). "The Bariatric Quality of Life (BQL) Index: A Measure of Well-being in Obesity Surgery Patients." Obesity Surgery **15**(4): 538–545.

Background: Quality of life (QoL) is considered to be the true measure for the effectiveness of a surgical procedure, but there are only a few validated instruments available for bariatric surgery. Therefore, a new diseasespecific 30-item instrument was created, which was called Bariatric Quality of Life (BQL) questionnaire. Methods: To validate the BQL, we studied 133 patients after 4 different types of bariatric surgery. Initially, mean body mass index (BMI) was 47.2 ±7.6 kg/m2 and mean age was 38.8 ±11.0 years. At baseline, and 1, 6, and 12 months after surgery, patients filled in the BQL, the SF-12 (Short Form of SF-36 Health Survey), the GIQLI (Gastrointestinal Quality of Life Index), and the BAROS (Bariatric Analysis and Reporting Outcome System). Results: Internal consistency of the BQL was found to be good, with Cronbach's alpha ranging between 0.71 and 0.86. Factor analyses suggested that the BQL included a highly consistent set of QoL items and a second part on co-morbidities and gastrointestinal symptoms. At the 12 months follow-up, the BQL was closely correlated to SF 12 (Pearson's r = 0.86), GIQLI (0.68), BAROS (0.71), and excess weight loss (0.55). Standardized effect sizes over time were larger for the BQL (1.39 and 1.58) than for the other instruments. Conclusions: The BQL questionnaire is a validated instrument ready for clinical use.

Weiner, S., et al. (2009). "Validation of the adapted Bariatric Quality of Life Index (BQL) in a prospective study in 446 bariatric patients as one-factor model." Obesity Facts **2 Suppl 1**(Suppl 1): 63–66.

BACKGROUND: The Bariatric Quality of Life Index (BQL) was created and validated as a nine-factor model in 2005 for the measurement of quality of life (QoL) in patients before and after bariatric surgery.Even though the results were acceptable, the statistical structure of the test was very unclear. METHODS: A total of 466 patients were enrolled in an ongoing prospective longitudinal German study. The assessment took place preoperatively and at 1, 3,6, 9, and 12 months postoperatively. After that period, reevaluations were done on a yearly basis. In addition to demographic and clinical data, QoL data were collected using the BQL, the Short Form 12 (SF-12v2), the Gastrointestinal Quality of Life Index(GIQLI), and the Bariatric Analysis and Reporting Outcome System(BAROS; old version since the study started in 2001). Statistical parameters for contingency (Cronbach's a), construct and criterion validity (Pearson's r), and responsiveness (standardized effect sizes) were calculated. The data of the assessments conducted preoperatively and after 6 and 12 months were used for the validation. RESULTS: The factor analysis and the screeplot showed that a one-factor solution explained 45.37% of variance. The selectivity of the items ranged between 0.61 and 0.85, and Cronbach's a was 0.898. The measurements showed similar excellent results with the analysis of all measurement points. Pearson's test showed a good retest reliability (r = 0.9). The correlations with the SF-12 and the Moorehead-Ardelt I questionnaire(MA-I) were significant, while the correlation with the GIQLI was low. The results of the correlation with the excess weight loss(EWL) (0.45 and 0.49) and the BMI (-0.38 and -0.47) were good. CONCLUSION: The BQL is a valid instrument and should be preferred over generic questionnaires as it provides better responsiveness.

World Health Organization (2020). WHO Director-General's opening remarks at the media briefing on COVID-19 - 11 March 2020. Geneva, World Health Organization.
