# Supplementary material for: The Impact of the COVID-19 Pandemic on Weight Loss and Quality of Life One Year Post Metabolic Bariatric Surgery
Source: Obes Surg. 2026 Mar 9;36(4):1503–9. doi: 10.1007/s11695-026-08515-6 (PMC13083447; doi:10.1007/s11695-026-08515-6)
Supplement: Supplementary file 2 — Supplementary Material 2 (HTML 1.08 MB) [file 11695_2026_8515_MOESM2_ESM.html]

Supplementary R - Code


# Supplementary R - Code

# 1 Information

This document describes the R-Code that was used to prepare and
analyze the data for the following paper:

**Impact of the COVID-19 pandemic on weight loss and quality of
life one year post bariatric surgery**

**Authors:** Michael Hanselmann, Sophie Merzweiler

# 2 Preparatory steps

First several packages were installed and loaded:

```
### Load the required packages 
library(rmarkdown)
library(epitools)
library(readxl)
library(rlang)
library(stringi)
library(tidyverse)
library(dplyr)
library(lubridate)
library(table1)
library(openxlsx)
library(flextable)
library(psych)
library(VIM)
library(gtsummary)

### Set Seed to make results reproducible 
set.seed(1234567)
```

# 3 Data preparation

**Study population:** The study population consisted of
patients who underwent gastric bypass (proximal and distal) or sleeve
gastrectomy as primary treatment for severe obesity between 1 December
2018 and 28 February 2019 (No-COVID-19 group) or between 1 December 2019
and 29 February 2020 (COVID-19 group) in a bariatric clinic in Germany.
We used prospectively collected data from the German register for
obesity and metabolic surgery StuDoQ|Metabolische und bariatrische
Erkrankungen (StuDoQ|MBE) (for more information visit http://www.dgav.de/studoq/studoqmbe.html). Certified
centers for metabolic and bariatric surgery or centers seeking
certification throughout Germany submit the clinical data of patients
with metabolic and bariatric diseases who have undergone surgery to the
Study, Documentation and Quality Centre (StuDoQ) of the German Society
for General and Visceral Surgery (DGAV). The data is documented in the
StuDoQ|MBE database (Deutsche Gesellschaft für Allgemein- und
Viszeralchirurgie e.V, 2023). Data collection for each participant is
done routinely during the examinations in the bariatric clinic.

## 3.1 Create initial data set

The original data set was retrieved from STUDOQ | Metabolische und
bariatrische Erkrankungen and included data for patients that underwent
gastric bypass (proximal and distal) or sleeve gastrectomy as primary
treatment for severe obesity between 2018-01-01 and 2022-12-31 (n=
57.010).

```
### Loading the original data set:
STUDOQ_original <- read_xlsx("Daten StuDoQ ACHT.xlsx")


### Create data set to work with that only includes variables that are neede to conduct the planned analyses

STUDOQ <- dplyr::select(STUDOQ_original, sex, dob, opdate, optechnique, graduation, employment,
                        app_diabetes_ii, fu2_diabetes_ii, app_hypertonie, fu2_hypertonie,  app_dyslipidaemie, app_apnoe,
                        height, preop_current_weight, fu1_weight, fu2_weight, fu1_completed, fu2_completed, fu2_bql_01, fu2_bql_02,fu2_bql_03,
                        fu2_bql_04, fu2_bql_05,fu2_bql_06, fu2_bql_07,fu2_bql_08, fu2_bql_09, fu2_bql_10a, fu2_bql_10b,
                        fu2_bql_10c, fu2_bql_11,   fu2_validate_postopcomplic,
                        fu2_postop_gastricband_portinfection,
                        fu2_postop_gastricband_bandslippage,
                        fu2_postop_gastricband_bandmigration,
                        fu2_postop_gastricband_other, pseudo_klinik_id)
```

## 3.2 Create and transform variables

The code below creates and transforms the outcomes and covariables
that are needed for the planned analyses step by step.

### 3.2.1 Rename Variables/Levels

The classes (e.g. character, numeric etc.) of the variables were
checked and changed if necessary. - Variables with the data type
“character” are transformed into data type “factor”, since they are
categorical answers. - The variables “opdate” and “date of birth” are
transformed into class “Date”. - Variables are renamed.

```
### check types of variables
str(STUDOQ)
```

```
## tibble [57,010 × 37] (S3: tbl_df/tbl/data.frame)
##  $ sex                                 : chr [1:57010] "weiblich" "weiblich" "weiblich" "weiblich" ...
##  $ dob                                 : POSIXct[1:57010], format: "1971-01-31" "1970-06-30" ...
##  $ opdate                              : POSIXct[1:57010], format: "2019-08-14" "2019-05-22" ...
##  $ optechnique                         : chr [1:57010] "Sleeve Gastrectomy (SG)" "Sleeve Gastrectomy (SG)" "Sleeve Gastrectomy (SG)" "Sleeve Gastrectomy (SG)" ...
##  $ graduation                          : chr [1:57010] "Hochschulreife" "Realschule / Mittlere Reife" "Realschule / Mittlere Reife" "Realschule / Mittlere Reife" ...
##  $ employment                          : chr [1:57010] "Vollzeit (>= 35 h/Woche)" "nicht erwerbstätig" "arbeitsunfähig" "Vollzeit (>= 35 h/Woche)" ...
##  $ app_diabetes_ii                     : chr [1:57010] "Nein" "Ja" "Ja" "Nein" ...
##  $ fu2_diabetes_ii                     : chr [1:57010] "Nein" "Nein" "k. A." "Nein" ...
##  $ app_hypertonie                      : chr [1:57010] "Nein" "Ja" "Ja" "Ja" ...
##  $ fu2_hypertonie                      : chr [1:57010] "Nein" "Nein" "k. A." "Ja,verbessert" ...
##  $ app_dyslipidaemie                   : chr [1:57010] "Nein" "Nein" "Nein" "Nein" ...
##  $ app_apnoe                           : chr [1:57010] "Nein" "Nein" "Ja" "Nein" ...
##  $ height                              : num [1:57010] 153 163 168 158 178 177 160 174 185 168 ...
##  $ preop_current_weight                : num [1:57010] 105 142 168 128 182 165 164 147 168 129 ...
##  $ fu1_weight                          : num [1:57010] 89 115 132 111 144 118 141 126 NA 106 ...
##  $ fu2_weight                          : num [1:57010] 82 98 NA 82 98 96 120 NA NA NA ...
##  $ fu1_completed                       : chr [1:57010] "Ja" "Ja" "Ja" "Ja" ...
##  $ fu2_completed                       : chr [1:57010] "Ja, 1. Jahr" "Ja, 1. Jahr" "k. A." "Ja, 1. Jahr" ...
##  $ fu2_bql_01                          : chr [1:57010] "5 stimmt vollkommen" "2 stimmt eher nicht" "k. A." "k. A." ...
##  $ fu2_bql_02                          : chr [1:57010] "5 stimmt vollkommen" "2 stimmt eher nicht" "k. A." "k. A." ...
##  $ fu2_bql_03                          : chr [1:57010] "5 sehr gut" "5 sehr gut" "k. A." "k. A." ...
##  $ fu2_bql_04                          : chr [1:57010] "5 stimmt vollkommen" "3 teils / teils" "k. A." "k. A." ...
##  $ fu2_bql_05                          : chr [1:57010] "5 stimmt vollkommen" "3 teils / teils" "k. A." "k. A." ...
##  $ fu2_bql_06                          : chr [1:57010] "1 stimmt überhaupt nicht" "2 stimmt eher nicht" "k. A." "k. A." ...
##  $ fu2_bql_07                          : chr [1:57010] "1 stimmt überhaupt nicht" "2 stimmt eher nicht" "k. A." "k. A." ...
##  $ fu2_bql_08                          : chr [1:57010] "1 stimmt überhaupt nicht" "1 stimmt überhaupt nicht" "k. A." "k. A." ...
##  $ fu2_bql_09                          : chr [1:57010] "5 stimmt vollkommen" "5 stimmt vollkommen" "k. A." "k. A." ...
##  $ fu2_bql_10a                         : chr [1:57010] "1 stimmt überhaupt nicht" "2 stimmt eher nicht" "k. A." "k. A." ...
##  $ fu2_bql_10b                         : chr [1:57010] "1 stimmt überhaupt nicht" "2 stimmt eher nicht" "k. A." "k. A." ...
##  $ fu2_bql_10c                         : chr [1:57010] "1 stimmt überhaupt nicht" "2 stimmt eher nicht" "k. A." "k. A." ...
##  $ fu2_bql_11                          : chr [1:57010] "5 stimmt vollkommen" "4 stimmt eher" "k. A." "k. A." ...
##  $ fu2_validate_postopcomplic          : chr [1:57010] "Nein" "Nein" "k. A." "Nein" ...
##  $ fu2_postop_gastricband_portinfection: chr [1:57010] "Nein" "Nein" "k. A." "Nein" ...
##  $ fu2_postop_gastricband_bandslippage : chr [1:57010] "Nein" "Nein" "k. A." "Nein" ...
##  $ fu2_postop_gastricband_bandmigration: chr [1:57010] "Nein" "Nein" "k. A." "Nein" ...
##  $ fu2_postop_gastricband_other        : chr [1:57010] "Nein" "Nein" "k. A." "Nein" ...
##  $ pseudo_klinik_id                    : chr [1:57010] "53289" "53289" "53289" "53289" ...
```

```
### change data type character into data type factor 
STUDOQ <- mutate(STUDOQ, sex = as.factor(sex),
                         optechnique = as.factor(optechnique),
                         graduation = as.factor(graduation),
                         employment = as.factor(employment),
                         app_diabetes_ii = as.factor(app_diabetes_ii),
                         app_hypertonie = as.factor(app_hypertonie),
                         app_dyslipidaemie = as.factor(app_dyslipidaemie),
                         app_apnoe = as.factor(app_apnoe),
                         fu1_completed = as.factor(fu1_completed),
                         fu2_completed = as.factor(fu2_completed),
                         fu2_bql_01 = as.factor(fu2_bql_01),
                         fu2_bql_02 = as.factor(fu2_bql_02),
                         fu2_bql_03 = as.factor(fu2_bql_03),
                         fu2_bql_04 = as.factor(fu2_bql_04),
                         fu2_bql_05 = as.factor(fu2_bql_05),
                         fu2_bql_06 = as.factor(fu2_bql_06),
                         fu2_bql_07 = as.factor(fu2_bql_07),
                         fu2_bql_08 = as.factor(fu2_bql_08),
                         fu2_bql_09 = as.factor(fu2_bql_09),
                         fu2_bql_10a = as.factor(fu2_bql_10a),
                         fu2_bql_10b = as.factor(fu2_bql_10b),
                         fu2_bql_10c = as.factor(fu2_bql_10c),
                         fu2_bql_11 = as.factor(fu2_bql_11),
                         fu2_diabetes_ii = as.factor(fu2_diabetes_ii),
                         fu2_hypertonie = as.factor(fu2_hypertonie),
                         fu2_validate_postopcomplic = as.factor(fu2_validate_postopcomplic),
                         fu2_postop_gastricband_portinfection = as.factor(fu2_postop_gastricband_portinfection),
                         fu2_postop_gastricband_bandslippage = as.factor(fu2_postop_gastricband_bandslippage),
                         fu2_postop_gastricband_bandmigration = as.factor(fu2_postop_gastricband_bandmigration),
                         fu2_postop_gastricband_other = as.factor(fu2_postop_gastricband_other))

### change variable opdate and date of birth into class "Date" representing calendar dates
STUDOQ <- mutate(STUDOQ, opdate = as.Date(opdate))
STUDOQ <- mutate(STUDOQ, dob = as.Date(dob))

### Rename Variables
STUDOQ <- rename(STUDOQ, weight_follow_up = fu2_weight,
                 weight_3month = fu1_weight,
                 op_date = opdate,
                 date_of_birth = dob,
                 weight_baseline = preop_current_weight,
                 diabetes_2_baseline = app_diabetes_ii,
                 hypertension_baseline = app_hypertonie,
                 sleep_apnoea_baseline = app_apnoe,
                 dyslipidemia_baseline = app_dyslipidaemie,
                 diabetes_2_follow_up = fu2_diabetes_ii,
                 hypertension_follow_up = fu2_hypertonie,
                 bql_01 = fu2_bql_01,
                 bql_02 = fu2_bql_02,
                 bql_03 = fu2_bql_03,
                 bql_04 = fu2_bql_04,
                 bql_05 = fu2_bql_05,
                 bql_06 = fu2_bql_06,
                 bql_07 = fu2_bql_07,
                 bql_08 = fu2_bql_08,
                 bql_09 = fu2_bql_09,
                 bql_10 = fu2_bql_10a,
                 bql_11 = fu2_bql_10b,
                 bql_12 = fu2_bql_10c,
                 bql_13 = fu2_bql_11,
                 new_complications_follow_up = fu2_validate_postopcomplic,
                 portinfection = fu2_postop_gastricband_portinfection,
                 bandslippage = fu2_postop_gastricband_bandslippage,
                 bandmigration = fu2_postop_gastricband_bandmigration,
                 other_complications = fu2_postop_gastricband_other)
```

### 3.2.2 Create addtitional baseline characteristics

Three new variables “Study group”, “Age”, “BMI” were created:

1. **Study group:**

- COVID-19 group: December 2019 - February 2020
- No-COVID-19 group: December 2018 - February 2019

2. **Age:** Age at time of surgery was calculated. 52
   weeks were selected to calculate age in years and then rounded.
3. **BMI:** BMI was calculated as follows: BMI =
   (weight\_baseline / (height/100)^2)) and then rounded.

```
### Create Variable "Study group"

STUDOQ <- STUDOQ %>% mutate(studygroup =  case_when(
                                (month(op_date) %in% c(12) & year(op_date) %in% c(2019)) |
                                  (month(op_date) %in% c(1, 2) & year(op_date) %in% c(2020)) ~ "COVID-19",
                                (month(op_date) %in% c(12) & year(op_date) %in% c(2018)) |
                                  (month(op_date) %in% c(1, 2) & year(op_date) %in% c(2019)) ~ "No-COVID-19",
                                TRUE ~ "NP")) %>%
                                mutate(studygroup = as.factor(studygroup)) %>% 
                                mutate(studygroup = fct_relevel(studygroup, c("No-COVID-19", "COVID-19")))

### Create Variable Age
# 52 weeks were chosen to use for the calculation for age in years

STUDOQ$age_weeks <- difftime(STUDOQ$op_date, STUDOQ$date_of_birth, units = "weeks")
STUDOQ <- transform(STUDOQ, age_years = (age_weeks/52))
STUDOQ <- mutate(STUDOQ, age_years = as.numeric(age_years))
STUDOQ$age_years <- round(STUDOQ$age_years)
 
### Create Variable BMI
STUDOQ <- transform(STUDOQ, BMI = ( weight_baseline / (height/100)^2))
STUDOQ$BMI <- round(STUDOQ$BMI)
```

### 3.2.3 Apply eligibility criteria

Eligibility criteria for all included patients were: Age, informed
consent, bariatric-metabolic operation (gastric bypass (proximal and
distal) or sleeve gastrectomy) at a clinic in Germany, recording of
patient data within the framework of StuDoQ|MBE, time point of
surgery.

The following eligibility criteria were applied:

- declaration of consent = yes -> Has already been applied by the
  provider of the original data set
- optechnique: Proximal & Distal Roux-en-y gastric bypass (RYGB),
  Sleeve gastrectomy (SG) -> Has already been applied by the provider
  of the original data set
- clinic in Germany = yes -> only data from clinics in Germany is
  available in the original dataset
- opdate: >= “2018-12-01” & opdate <= “2019-02-28” | >=
  “2019-12-01” & opdate <= “2020-02-29”
- age ≥ 18

#### 3.2.3.1 OP Date

```
### Dismiss all observations whose op dates were not in the defined time period
STUDOQ <- filter(STUDOQ, studygroup != "NP")
```

#### 3.2.3.2 Age

```
### Exploring how many observations have age <18
nrow(subset(STUDOQ, age_years <18))
```

```
## [1] 6
```

```
# Comment:6 patients have age <18

### applying exclusion criteria <18 
STUDOQ <- filter(STUDOQ, age_years >= 18 | NA )
```

### 3.2.4 Translation

Dataset was translated from **German** to
**English**.

#### 3.2.4.1 Covariables

For some categorical variables certain categories are
**grouped** together in the english version:

- **operation technique:** proximal and distal gastric
  bypass are grouped to RYGB
- **employment:**

  - “employed” entails “part-” and “full time”
  - “not employed” entails “housewife” and “not employed”
  - “unknown” entails “unknown” and “other”
- **graduation:** classified as low-intermediate-high,
  without, unknown, other

  - low = Hauptschule / Volksschule
  - intermediate = Realschule / Mittlere Reife
  - high = Hochschulreife (A-levels)
  - “unknown” entails “unknown” and “other”
- **OSA:**

  - “Ja, mit CPAP-Beatmung” coded as “yes”
- The category “k. A.” was always recoded as “NA”.

```
### sex
summary(STUDOQ$sex)
```

```
##     k.A. männlich weiblich 
##        2     1715     4142
```

```
STUDOQ <- STUDOQ %>%
  mutate(sex = recode(sex, "männlich" = "male", "weiblich" = "female"))
STUDOQ$sex[STUDOQ$sex == "k. A."] <- NA
STUDOQ$sex <- droplevels(STUDOQ$sex, exclude = "k.A.")
summary(STUDOQ$sex)
```

```
##   male female   NA's 
##   1715   4142      2
```

```
### Surgical Technique
summary(STUDOQ$optechnique)
```

```
##   distaler Roux-en-y-Magenbypass (RYGB) proximaler Roux-en-y-Magenbypass (RYGB) 
##                                      39                                    2160 
##                 Sleeve Gastrectomy (SG) 
##                                    3660
```

```
STUDOQ <- STUDOQ %>%
  mutate(optechnique = recode(optechnique, "proximaler Roux-en-y-Magenbypass (RYGB)" = "Bypass", "distaler Roux-en-y-Magenbypass (RYGB)" = "Bypass", "Sleeve Gastrectomy (SG)" = "Sleeve"))
STUDOQ$optechnique[STUDOQ$optechnique == "k. A."] <- NA
STUDOQ$optechnique <- droplevels(STUDOQ$optechnique, exclude = "k. A.")
summary(STUDOQ$optechnique)
```

```
## Bypass Sleeve 
##   2199   3660
```

```
### employment (grouped)
summary(STUDOQ$employment)
```

```
##                     andere             arbeitsunfähig 
##                        150                        160 
##                   berentet          Hausfrau/Hausmann 
##                        335                        337 
##                      k. A.         nicht erwerbstätig 
##                       1438                        552 
##     Teilzeit (<15 h/Woche) Teilzeit (15 - 35 h/Woche) 
##                        149                        589 
##                  Unbekannt   Vollzeit (>= 35 h/Woche) 
##                        429                       1720
```

```
STUDOQ <- STUDOQ %>%
  mutate(employment = recode(employment, "arbeitsunfähig" = "not employed", "berentet" = "retired", "Hausfrau/Hausmann" = "housewife/ househusband", "nicht erwerbstätig" = "not employed", "Teilzeit (<15 h/Woche)" = "employed", "Teilzeit (15 - 35 h/Woche)" = "employed", "Vollzeit (>= 35 h/Woche)" = "employed", "Unbekannt" = "unknown", "andere" = "unknown"))
STUDOQ$employment[STUDOQ$employment == "k. A."] <- NA
STUDOQ$employment <- droplevels(STUDOQ$employment, exclude = "k. A.")
summary(STUDOQ$employment)
```

```
##                 unknown            not employed                 retired 
##                     579                     712                     335 
## housewife/ househusband                employed                    <NA> 
##                     337                    2458                    1438
```

```
### Graduation
summary(STUDOQ$graduation)
```

```
##                     anderer   Hauptschule / Volksschule 
##                         139                        1149 
##              Hochschulreife                       k. A. 
##                         674                        1443 
##              ohne Abschluss Realschule / Mittlere Reife 
##                         183                        1480 
##                   Unbekannt 
##                         791
```

```
STUDOQ <- STUDOQ %>%
  mutate(graduation = recode(graduation, "Hochschulreife" = "high", "Realschule / Mittlere Reife" = "intermediate", "Hauptschule / Volksschule" = "low", "ohne Abschluss" = "without", "anderer" = "unknown", "Unbekannt" = "unknown"))
STUDOQ$graduation[STUDOQ$graduation == "k. A."] <- NA
STUDOQ$graduation <- droplevels(STUDOQ$graduation, exclude = "k. A.")
summary(STUDOQ$graduation)
```

```
##      unknown          low         high      without intermediate         <NA> 
##          930         1149          674          183         1480         1443
```

```
### T2D Baseline
summary(STUDOQ$diabetes_2_baseline)
```

```
##    Ja k. A.  Nein 
##   608  3488  1763
```

```
STUDOQ <- STUDOQ %>%
  mutate(diabetes_2_baseline = recode(diabetes_2_baseline, "Ja" = "yes", "Nein" = "no"))
STUDOQ$diabetes_2_baseline[STUDOQ$diabetes_2_baseline == "k. A."] <- NA
STUDOQ$diabetes_2_baseline <- droplevels(STUDOQ$diabetes_2_baseline, exclude = "k. A.")
summary(STUDOQ$diabetes_2_baseline)
```

```
##  yes   no <NA> 
##  608 1763 3488
```

```
### Hypertension
summary(STUDOQ$hypertension_baseline)
```

```
##    Ja k. A.  Nein 
##  1363  3488  1008
```

```
STUDOQ <- STUDOQ %>%
  mutate(hypertension_baseline = recode(hypertension_baseline, "Ja" = "yes", "Nein" = "no"))
STUDOQ$hypertension_baseline[STUDOQ$hypertension_baseline == "k. A."] <- NA
STUDOQ$hypertension_baseline <- droplevels(STUDOQ$hypertension_baseline, exclude = "k. A.")
summary(STUDOQ$hypertension_baseline)
```

```
##  yes   no <NA> 
## 1363 1008 3488
```

```
### Sleep apnoea baseline
summary(STUDOQ$sleep_apnoea_baseline)
```

```
##                    Ja Ja, mit CPAP-Beatmung                 k. A. 
##                   475                   333                  3488 
##                  Nein 
##                  1563
```

```
STUDOQ <- STUDOQ %>%
  mutate(sleep_apnoea_baseline = recode(sleep_apnoea_baseline, "Ja" = "yes", "Ja, mit CPAP-Beatmung" = "yes" ,"Nein" = "no"))
STUDOQ$sleep_apnoea_baseline[STUDOQ$sleep_apnoea_baseline == "k. A."] <- NA
STUDOQ$sleep_apnoea_baseline <- droplevels(STUDOQ$sleep_apnoea_baseline, exclude = "k. A.")
summary(STUDOQ$sleep_apnoea_baseline)
```

```
##  yes   no <NA> 
##  808 1563 3488
```

```
### dyslipidemia
summary(STUDOQ$dyslipidemia_baseline)
```

```
##    Ja k. A.  Nein 
##   446  3488  1925
```

```
STUDOQ <- STUDOQ %>%
  mutate(dyslipidemia_baseline = recode(dyslipidemia_baseline, "Ja" = "yes", "Nein" = "no"))
STUDOQ$dyslipidemia_baseline[STUDOQ$dyslipidemia_baseline == "k. A."] <- NA
STUDOQ$dyslipidemia_baseline <- droplevels(STUDOQ$dyslipidemia_baseline, exclude = "k. A.")
summary(STUDOQ$dyslipidemia_baseline)
```

```
##  yes   no <NA> 
##  446 1925 3488
```

#### 3.2.4.2 BQL outcome

- **Questions 1-5 and question 9, 11 of BQL
  questionnaire:** equivalent to bql\_01- bql\_05, bql\_09, bql\_13:
  Categories are recoded into likert scale level 1-5.
- **Questions 6, 7, 8, 10a, 10b, 10c:** The likert
  scale is inverted for the corresponding items BQL\_06, BQL\_07, BQL\_08,
  BQL\_10, BQL\_11, BQL\_12

  - 1 >> 5 need to be inverted
  - 2 >> 4
  - 3 >> 3
  - 4 >> 2
  - 5 >> 1
- **Question 10b:** categorical answer “nicht
  berufstätig” = “not employed” is translated and replaced with the mean
  value of the other observations.
- The category “k. A.” is always recoded as “NA”.

```
### BQL

## 1
summary(STUDOQ$bql_01)
```

```
## 1 stimmt überhaupt nicht      2 stimmt eher nicht          3 teils / teils 
##                       56                      124                      363 
##            4 stimmt eher      5 stimmt vollkommen                    k. A. 
##                      335                      341                      857 
##                     NA's 
##                     3783
```

```
STUDOQ <- STUDOQ %>%
  mutate(bql_01 = recode(bql_01, "1 stimmt überhaupt nicht" = "1","2 stimmt eher nicht" = "2", "3 teils / teils" = "3", "4 stimmt eher" = "4", "5 stimmt vollkommen" = "5"))
STUDOQ$bql_01[STUDOQ$bql_01 == "k. A."] <- NA
STUDOQ$bql_01 <- droplevels(STUDOQ$bql_01, exclude = "k. A.")
summary(STUDOQ$bql_01)
```

```
##    1    2    3    4    5 <NA> 
##   56  124  363  335  341 4640
```

```
## 2
summary(STUDOQ$bql_02)
```

```
## 1 stimmt überhaupt nicht      2 stimmt eher nicht          3 teils / teils 
##                       74                      135                      286 
##            4 stimmt eher      5 stimmt vollkommen                    k. A. 
##                      312                      412                      857 
##                     NA's 
##                     3783
```

```
STUDOQ <- STUDOQ %>%
  mutate(bql_02 = recode(bql_02, "1 stimmt überhaupt nicht" = "1","2 stimmt eher nicht" = "2", "3 teils / teils" = "3", "4 stimmt eher" = "4", "5 stimmt vollkommen" = "5"))
STUDOQ$bql_02[STUDOQ$bql_02 == "k. A."] <- NA
STUDOQ$bql_02 <- droplevels(STUDOQ$bql_02, exclude = "k. A.")
summary(STUDOQ$bql_02)
```

```
##    1    2    3    4    5 <NA> 
##   74  135  286  312  412 4640
```

```
## 3
summary(STUDOQ$bql_03)
```

```
##      1 schlecht   2 weniger gut 3 teils / teils           4 gut      5 sehr gut 
##              21              43             186             560             409 
##           k. A.            NA's 
##             857            3783
```

```
STUDOQ <- STUDOQ %>%
  mutate(bql_03 = recode(bql_03, "1 schlecht" = "1","2 weniger gut" = "2", "3 teils / teils" = "3", "4 gut" = "4", "5 sehr gut" = "5"))
STUDOQ$bql_03[STUDOQ$bql_03 == "k. A."] <- NA
STUDOQ$bql_03 <- droplevels(STUDOQ$bql_03, exclude = "k. A.")
summary(STUDOQ$bql_03)
```

```
##    1    2    3    4    5 <NA> 
##   21   43  186  560  409 4640
```

```
## 4
summary(STUDOQ$bql_04)
```

```
## 1 stimmt überhaupt nicht      2 stimmt eher nicht          3 teils / teils 
##                      102                       75                      218 
##            4 stimmt eher      5 stimmt vollkommen                    k. A. 
##                      281                      543                      857 
##                     NA's 
##                     3783
```

```
STUDOQ <- STUDOQ %>%
  mutate(bql_04 = recode(bql_04, "1 stimmt überhaupt nicht" = "1","2 stimmt eher nicht" = "2", "3 teils / teils" = "3", "4 stimmt eher" = "4", "5 stimmt vollkommen" = "5"))
STUDOQ$bql_04[STUDOQ$bql_04 == "k. A."] <- NA
STUDOQ$bql_04 <- droplevels(STUDOQ$bql_04, exclude = "k. A.")
summary(STUDOQ$bql_04)
```

```
##    1    2    3    4    5 <NA> 
##  102   75  218  281  543 4640
```

```
## 5
summary(STUDOQ$bql_05)
```

```
## 1 stimmt überhaupt nicht      2 stimmt eher nicht          3 teils / teils 
##                       58                       57                      186 
##            4 stimmt eher      5 stimmt vollkommen                    k. A. 
##                      294                      623                      858 
##                     NA's 
##                     3783
```

```
STUDOQ <- STUDOQ %>%
  mutate(bql_05 = recode(bql_05, "1 stimmt überhaupt nicht" = "1","2 stimmt eher nicht" = "2", "3 teils / teils" = "3", "4 stimmt eher" = "4", "5 stimmt vollkommen" = "5"))
STUDOQ$bql_05[STUDOQ$bql_05 == "k. A."] <- NA
STUDOQ$bql_05 <- droplevels(STUDOQ$bql_05, exclude = "k. A.")
summary(STUDOQ$bql_05)
```

```
##    1    2    3    4    5 <NA> 
##   58   57  186  294  623 4641
```

```
## 6 (inverted likert scale)
summary(STUDOQ$bql_06)
```

```
## 1 stimmt überhaupt nicht      2 stimmt eher nicht          3 teils / teils 
##                      782                      233                       95 
##            4 stimmt eher      5 stimmt vollkommen                    k. A. 
##                       45                       64                      857 
##                     NA's 
##                     3783
```

```
STUDOQ <- STUDOQ %>%
  mutate(bql_06 = recode(bql_06, "1 stimmt überhaupt nicht" = "5","2 stimmt eher nicht" = "4", "3 teils / teils" = "3", "4 stimmt eher" = "2", "5 stimmt vollkommen" = "1"))
STUDOQ$bql_06[STUDOQ$bql_06 == "k. A."] <- NA
STUDOQ$bql_06 <- droplevels(STUDOQ$bql_06, exclude = "k. A.")
summary(STUDOQ$bql_06)
```

```
##    5    4    3    2    1 <NA> 
##  782  233   95   45   64 4640
```

```
## 7 (inverted likert scale) 
summary(STUDOQ$bql_07)
```

```
## 1 stimmt überhaupt nicht      2 stimmt eher nicht          3 teils / teils 
##                      636                      251                      226 
##            4 stimmt eher      5 stimmt vollkommen                    k. A. 
##                       67                       39                      857 
##                     NA's 
##                     3783
```

```
STUDOQ <- STUDOQ %>%
  mutate(bql_07 = recode(bql_07, "1 stimmt überhaupt nicht" = "5","2 stimmt eher nicht" = "4", "3 teils / teils" = "3", "4 stimmt eher" = "2", "5 stimmt vollkommen" = "1"))
STUDOQ$bql_07[STUDOQ$bql_07 == "k. A."] <- NA
STUDOQ$bql_07 <- droplevels(STUDOQ$bql_07, exclude = "k. A.")
summary(STUDOQ$bql_07)
```

```
##    5    4    3    2    1 <NA> 
##  636  251  226   67   39 4640
```

```
## 8 (inverted likert scale) 
summary(STUDOQ$bql_08)
```

```
## 1 stimmt überhaupt nicht      2 stimmt eher nicht          3 teils / teils 
##                      642                      218                      206 
##            4 stimmt eher      5 stimmt vollkommen                    k. A. 
##                       84                       69                      857 
##                     NA's 
##                     3783
```

```
STUDOQ <- STUDOQ %>%
  mutate(bql_08 = recode(bql_08, "1 stimmt überhaupt nicht" = "5","2 stimmt eher nicht" = "4", "3 teils / teils" = "3", "4 stimmt eher" = "2", "5 stimmt vollkommen" = "1"))
STUDOQ$bql_08[STUDOQ$bql_08 == "k. A."] <- NA
STUDOQ$bql_08 <- droplevels(STUDOQ$bql_08, exclude = "k. A.")
summary(STUDOQ$bql_08)
```

```
##    5    4    3    2    1 <NA> 
##  642  218  206   84   69 4640
```

```
## 9 
summary(STUDOQ$bql_09)
```

```
## 1 stimmt überhaupt nicht      2 stimmt eher nicht          3 teils / teils 
##                       48                       44                      225 
##            4 stimmt eher      5 stimmt vollkommen                    k. A. 
##                      434                      468                      857 
##                     NA's 
##                     3783
```

```
STUDOQ <- STUDOQ %>%
  mutate(bql_09 = recode(bql_09, "1 stimmt überhaupt nicht" = "1","2 stimmt eher nicht" = "2", "3 teils / teils" = "3", "4 stimmt eher" = "4", "5 stimmt vollkommen" = "5"))
STUDOQ$bql_09[STUDOQ$bql_09 == "k. A."] <- NA
STUDOQ$bql_09 <- droplevels(STUDOQ$bql_09, exclude = "k. A.")
summary(STUDOQ$bql_09)
```

```
##    1    2    3    4    5 <NA> 
##   48   44  225  434  468 4640
```

```
##  10 (inverted likert scale) 
summary(STUDOQ$bql_10)
```

```
## 1 stimmt überhaupt nicht      2 stimmt eher nicht          3 teils / teils 
##                      807                      180                      154 
##            4 stimmt eher      5 stimmt vollkommen                    k. A. 
##                       49                       29                      857 
##                     NA's 
##                     3783
```

```
STUDOQ <- STUDOQ %>%
  mutate(bql_10 = recode(bql_10, "1 stimmt überhaupt nicht" = "5","2 stimmt eher nicht" = "4", "3 teils / teils" = "3", "4 stimmt eher" = "2", "5 stimmt vollkommen" = "1"))
STUDOQ$bql_10[STUDOQ$bql_10 == "k. A."] <- NA
STUDOQ$bql_10 <- droplevels(STUDOQ$bql_10, exclude = "k. A.")
summary(STUDOQ$bql_10)
```

```
##    5    4    3    2    1 <NA> 
##  807  180  154   49   29 4640
```

```
## 11 (inverted likert scale, categorical answer "nicht berufstätig" = "not employed" was translated and then rplaced with mean value of the other observations)

summary(STUDOQ$bql_11)
```

```
## 1 stimmt überhaupt nicht      2 stimmt eher nicht          3 teils / teils 
##                      692                      123                       96 
##            4 stimmt eher      5 stimmt vollkommen                    k. A. 
##                       29                       33                      857 
##        nicht berufstätig                     NA's 
##                      246                     3783
```

```
STUDOQ <- STUDOQ %>%
  mutate(bql_11 = recode(bql_11, "1 stimmt überhaupt nicht" = "5","2 stimmt eher nicht" = "4", "3 teils / teils" = "3", "4 stimmt eher" = "2", "5 stimmt vollkommen" = "1", "nicht berufstätig" = "not employed"))
STUDOQ$bql_11[STUDOQ$bql_11 == "k. A."] <- NA
STUDOQ$bql_11 <- droplevels(STUDOQ$bql_11, exclude = "k. A.")
summary(STUDOQ$bql_11)
```

```
##            5            4            3            2            1 not employed 
##          692          123           96           29           33          246 
##         <NA> 
##         4640
```

```
# Convert factor variable to character to handle "not employed"
STUDOQ$bql_11 <- as.character(STUDOQ$bql_11)
class(STUDOQ$bql_11)
```

```
## [1] "character"
```

```
table(STUDOQ$bql_11)
```

```
## 
##            1            2            3            4            5 not employed 
##           33           29           96          123          692          246
```

```
summary(STUDOQ$bql_11)
```

```
##    Length     Class      Mode 
##      5859 character character
```

```
# Calculate the mean of ordinal values in variable x, handling missing values
ordinal_values <- as.numeric(STUDOQ$bql_11[!STUDOQ$bql_11 %in% "not employed" & !is.na(STUDOQ$bql_11)])
mean_value <- mean(ordinal_values, na.rm = TRUE)
summary(mean_value)
```

```
##    Min. 1st Qu.  Median    Mean 3rd Qu.    Max. 
##   4.451   4.451   4.451   4.451   4.451   4.451
```

```
rounded_mean_value <- round(mean_value)

# Replace "not employed"  values with the mean value
STUDOQ$bql_11[STUDOQ$bql_11 %in% c("not employed")] <- as.character(rounded_mean_value)

# Convert back to a factor
STUDOQ$bql_11 <- factor(STUDOQ$bql_11, levels = unique(STUDOQ$bql_11))
table(STUDOQ$bql_11)
```

```
## 
##   3   4   5   2   1 
##  96 369 692  29  33
```

```
# 12 (inverted likert scale) 
summary(STUDOQ$bql_12)
```

```
## 1 stimmt überhaupt nicht      2 stimmt eher nicht          3 teils / teils 
##                      758                      200                      163 
##            4 stimmt eher      5 stimmt vollkommen                    k. A. 
##                       61                       37                      857 
##                     NA's 
##                     3783
```

```
STUDOQ <- STUDOQ %>%
  mutate(bql_12 = recode(bql_12, "1 stimmt überhaupt nicht" = "5","2 stimmt eher nicht" = "4", "3 teils / teils" = "3", "4 stimmt eher" = "2", "5 stimmt vollkommen" = "1"))
STUDOQ$bql_12[STUDOQ$bql_12 == "k. A."] <- NA
STUDOQ$bql_12 <- droplevels(STUDOQ$bql_12, exclude = "k. A.")
summary(STUDOQ$bql_12)
```

```
##    5    4    3    2    1 <NA> 
##  758  200  163   61   37 4640
```

```
# 13
summary(STUDOQ$bql_13)
```

```
## 1 stimmt überhaupt nicht      2 stimmt eher nicht          3 teils / teils 
##                       92                       72                      271 
##            4 stimmt eher      5 stimmt vollkommen                    k. A. 
##                      369                      415                      857 
##                     NA's 
##                     3783
```

```
STUDOQ <- STUDOQ %>%
  mutate(bql_13 = recode(bql_13, "1 stimmt überhaupt nicht" = "1","2 stimmt eher nicht" = "2", "3 teils / teils" = "3", "4 stimmt eher" = "4", "5 stimmt vollkommen" = "5"))
STUDOQ$bql_13[STUDOQ$bql_13 == "k. A."] <- NA
STUDOQ$bql_13 <- droplevels(STUDOQ$bql_13, exclude = "k. A.")
summary(STUDOQ$bql_13)
```

```
##    1    2    3    4    5 <NA> 
##   92   72  271  369  415 4640
```

#### 3.2.4.3 Variables for Secondary Endpoints

**Type 2 Diabetes (T2D) and hypertension:** The
secondary outcomes T2D and hypertension are coded as binary outcomes
(yes/ no). The Yes-Category includes: *Yes, same as before surgery;
Yes, better than before surgery; Yes, worse than before surgery; Yes,
new occurrence*

**Participation at follow-up:**

- Participation at 3-month and 1-year follow-up appointment are coded
  as binary outcomes (yes/ no).
- The category “Kontakt verloren” (loss of follow up) is coded as
  no.
- In this step the categories “Ja, zwischenzeitliche Nachsorge” =
  “yes, interim care” and “Nachsorgerwechsel erfolgt” = “change of clinic”
  are translated

**Complications after 1-year:** Complications is coded
as binary outcome (yes/ no).

The category **“k. A.”** is always recoded as
**“NA”**.

```
### T2D at 1-year follow up
summary(STUDOQ$diabetes_2_follow_up)
```

```
## Ja,neu aufgetreten     Ja,unverändert      Ja,verbessert  Ja,verschlechtert 
##                  2                116                584                  4 
##              k. A.               Nein 
##               1967               3186
```

```
STUDOQ <- STUDOQ %>%
  mutate(diabetes_2_follow_up = recode(diabetes_2_follow_up, "Ja,neu aufgetreten" = "yes", "Ja,unverändert" = "yes", "Ja,verbessert" = "yes", "Ja,verschlechtert" = "yes", "Nein" = "no"))
STUDOQ$diabetes_2_follow_up[STUDOQ$diabetes_2_follow_up == "k. A."] <- NA
STUDOQ$diabetes_2_follow_up <- droplevels(STUDOQ$diabetes_2_follow_up, exclude = "k. A.")
summary(STUDOQ$diabetes_2_follow_up)
```

```
##  yes   no <NA> 
##  706 3186 1967
```

```
### Hypertension at 1-year follow up
summary(STUDOQ$hypertension_follow_up)
```

```
## Ja,neu aufgetreten     Ja,unverändert      Ja,verbessert  Ja,verschlechtert 
##                  4                412               1123                 19 
##              k. A.               Nein 
##               1967               2334
```

```
STUDOQ <- STUDOQ %>%
  mutate(hypertension_follow_up = recode(hypertension_follow_up, "Ja,neu aufgetreten" = "yes", "Ja,unverändert" = "yes", "Ja,verbessert" = "yes", "Ja,verschlechtert" = "yes", "Nein" = "no"))
STUDOQ$hypertension_follow_up[STUDOQ$hypertension_follow_up == "k. A."] <- NA
STUDOQ$hypertension_follow_up <- droplevels(STUDOQ$hypertension_follow_up, exclude = "k. A.")
summary(STUDOQ$hypertension_follow_up)
```

```
##  yes   no <NA> 
## 1558 2334 1967
```

```
### participation at 3-month follow-up appointment
summary(STUDOQ$fu1_completed)
```

```
##                                   Ja                                k. A. 
##                                 4541                                  726 
## Kontakt verloren (loss of follow up)            Nachsorgerwechsel erfolgt 
##                                  101                                   37 
##                                 Nein 
##                                  454
```

```
STUDOQ <- STUDOQ %>%
  mutate(fu1_completed = recode(fu1_completed, "Ja" = "yes", "Nein" = "no", "Nachsorgerwechsel erfolgt" = "change of clinic", "Kontakt verloren (loss of follow up)" = "no"))
STUDOQ$fu1_completed[STUDOQ$fu1_completed == "k. A."] <- NA
STUDOQ$fu1_completed <- droplevels(STUDOQ$fu1_completed, exclude = "k. A.")
summary(STUDOQ$fu1_completed)
```

```
##              yes               no change of clinic             <NA> 
##             4541              555               37              726
```

```
#

### participation at 1-year follow-up appointment
summary(STUDOQ$fu2_completed)
```

```
##                          Ja, 1. Jahr      Ja, zwischenzeitliche Nachsorge 
##                                 3742                                  136 
##                                k. A. Kontakt verloren (loss of follow up) 
##                                 1544                                  180 
##            Nachsorgerwechsel erfolgt                                 Nein 
##                                   66                                  191
```

```
STUDOQ <- STUDOQ %>%
  mutate(fu2_completed = recode(fu2_completed, "Ja, 1. Jahr" = "yes", "Ja, zwischenzeitliche Nachsorge" = "yes, interim care", "Nein" = "no", "Nachsorgerwechsel erfolgt" = "change of clinic", "Kontakt verloren (loss of follow up)" = "no"))
STUDOQ$fu2_completed[STUDOQ$fu2_completed == "k. A."] <- NA
STUDOQ$fu2_completed <- droplevels(STUDOQ$fu2_completed, exclude = "k. A.")
summary(STUDOQ$fu2_completed)
```

```
##               yes yes, interim care                no  change of clinic 
##              3742               136               371                66 
##              <NA> 
##              1544
```

### 3.2.5 Additional manipulations

#### 3.2.5.1 Recoding answers of participation at follow-up appointments

**3-month appointment:**

- “change of clinic”: If weight is available for the 3-month
  appointment and attendance at the 3-month appointment is the category
  “clinic change,” the response for attendance at the 3-month appointment
  is recoded to “yes” because the weight is measured and transmitted to
  the registry. If weight is not available, the observations are later
  recoded to NA.
- If weight is available for the 3-month appointment, particiaption
  3-month appointment is recoded from no to yes.
- “NA”: The category NA for participation in follow-up is recoded
  to “no” because no information was submitted to the registry. Therefore,
  it is assumed that these follow-up examinations did not take
  place.

**1-year follow-up appointment:**

- “yes, interim care”: The category “yes, interim care” is set to
  “yes” as data for 1-year follow-up appointment is present.
- “NA”: The category NA for participation in follow-up is recoded
  to “no” because no information was submitted to the registry. Therefore,
  it is assumed that these follow-up examinations did not take
  place.
- “change of clinic”: If weight is available for the 1-year
  appointment and attendance at the 1-year appointment has the category
  “clinic change,” the response for attendance at the 1-year appointment
  will be recoded to “yes” because the weight was measured and transmitted
  to the registry. If weight was not available, the observations were
  later recoded to NA.
- If weight was available for the 1-year appointment, participation
  1-year appointment was recoded from no to yes.

```
# Exploring subset interim_care
table(STUDOQ$fu2_completed)
```

```
## 
##               yes yes, interim care                no  change of clinic 
##              3742               136               371                66 
##              <NA> 
##              1544
```

```
# The category "yes, interim care" was recoded to "yes".
STUDOQ <- STUDOQ %>%
  mutate(fu2_completed = recode(fu2_completed, "yes, interim care" = "yes"))

summary(STUDOQ$fu2_completed)
```

```
##              yes               no change of clinic             NA's 
##             3878              371               66             1544
```

```
# If weight fu1 available >> recode FU1 particiaption from no to yes 
STUDOQ$fu1_completed[(STUDOQ$weight_3month != 'NA') & (STUDOQ$fu1_completed == 'no')] <- 'yes'

# If weight fu2 available >> recode FU2 participation to yes 
STUDOQ$fu2_completed[(STUDOQ$weight_follow_up != 'NA') & (STUDOQ$fu2_completed == 'no')] <- 'yes'

### Recoding change of clinic
# If two conditions were true: weight fu1 available and fu1_completed == 'change of clinic >> 3-month follow up was recoded to "yes" 
STUDOQ$fu1_completed[(STUDOQ$weight_3month != 'NA') & (STUDOQ$fu1_completed == 'change of clinic')] <- 'yes'

# If two conditions were true: weight fu1 available and fu2_completed == 'change of clinic >>  1-year follow up was recode to "yes" 
STUDOQ$fu2_completed[(STUDOQ$weight_follow_up != 'NA') & (STUDOQ$fu2_completed == 'change of clinic')] <- 'yes'

summary(STUDOQ$fu1_completed)
```

```
##              yes               no change of clinic             <NA> 
##             4594              518               21              726
```

```
summary(STUDOQ$fu2_completed)
```

```
##              yes               no change of clinic             NA's 
##             3899              361               55             1544
```

```
### convert NA to no
# Convert factor variable to character to handle "fu2"
STUDOQ$fu1_completed <- as.character(STUDOQ$fu1_completed)
STUDOQ$fu2_completed <- as.character(STUDOQ$fu2_completed)
class(STUDOQ$fu1_completed)
```

```
## [1] "character"
```

```
table(STUDOQ$fu1_completed)
```

```
## 
## change of clinic               no              yes 
##               21              518             4594
```

```
class(STUDOQ$fu2_completed)
```

```
## [1] "character"
```

```
table(STUDOQ$fu2_completed)
```

```
## 
## change of clinic               no              yes 
##               55              361             3899
```

```
STUDOQ$fu1_completed <- ifelse(is.na(STUDOQ$fu1_completed), "no", STUDOQ$fu1_completed)
STUDOQ$fu2_completed <- ifelse(is.na(STUDOQ$fu2_completed), "no", STUDOQ$fu2_completed)

table(STUDOQ$fu1_completed)
```

```
## 
## change of clinic               no              yes 
##               21             1244             4594
```

```
table(STUDOQ$fu2_completed)
```

```
## 
## change of clinic               no              yes 
##               55             1905             3899
```

```
### If there was no information about weight at follow-up appointment available in the registry, the participation at follow-up appointments were recoded to "NA" since we have the information that they changed clinics but do not know if they went to follow-up appointments or not.

STUDOQ$fu1_completed[STUDOQ$fu1_completed == "change of clinic"] <- NA
STUDOQ$fu2_completed[STUDOQ$fu2_completed == "change of clinic"] <- NA

table(STUDOQ$fu1_completed)
```

```
## 
##   no  yes 
## 1244 4594
```

```
table(STUDOQ$fu2_completed)
```

```
## 
##   no  yes 
## 1905 3899
```

```
# Convert character to factor variable 
STUDOQ$fu1_completed <- as.factor(STUDOQ$fu1_completed)
STUDOQ$fu2_completed <- as.factor(STUDOQ$fu2_completed)
```

#### 3.2.5.2 Calculation of primary outcomes

**%TWL**

- TWL is calculated as follows: TWL= (baseline weight – follow-up
  weight)
- %TWL is calculated as follows: 100% x (baseline weight - follow-up
  weight)/baseline weight

**BQL**

- The overall value of the BQL is determined. To do so, the mean of
  the Likert-scale (1-5) of the 13-item scale is calculated:

```
### TWL

# Calculating TWL 
STUDOQ$total_weight_loss <- (STUDOQ$weight_baseline - STUDOQ$weight_follow_up)
summary(STUDOQ$total_weight_loss)
```

```
##    Min. 1st Qu.  Median    Mean 3rd Qu.    Max.    NA's 
##  -53.00   33.00   43.00   43.81   54.00  126.00    1958
```

```
# Calculating %TWL
STUDOQ$total_weight_loss_percentage <- ((STUDOQ$weight_baseline - STUDOQ$weight_follow_up)/STUDOQ$weight_baseline)*100


### BQL

STUDOQ$bql_01_num[STUDOQ$bql_01 == "1"] <- 1
STUDOQ$bql_01_num[STUDOQ$bql_01 == "2"] <- 2
STUDOQ$bql_01_num[STUDOQ$bql_01 == "3"] <- 3
STUDOQ$bql_01_num[STUDOQ$bql_01 == "4"] <- 4
STUDOQ$bql_01_num[STUDOQ$bql_01 == "5"] <- 5
STUDOQ$bql_01_num[is.na(STUDOQ$bql_01)] <- NA


STUDOQ$bql_02_num[STUDOQ$bql_02 == "1"] <- 1
STUDOQ$bql_02_num[STUDOQ$bql_02 == "2"] <- 2
STUDOQ$bql_02_num[STUDOQ$bql_02 == "3"] <- 3
STUDOQ$bql_02_num[STUDOQ$bql_02 == "4"] <- 4
STUDOQ$bql_02_num[STUDOQ$bql_02 == "5"] <- 5
STUDOQ$bql_02_num[is.na(STUDOQ$bql_02)] <- NA

STUDOQ$bql_03_num[STUDOQ$bql_03 == "1"] <- 1
STUDOQ$bql_03_num[STUDOQ$bql_03 == "2"] <- 2
STUDOQ$bql_03_num[STUDOQ$bql_03 == "3"] <- 3
STUDOQ$bql_03_num[STUDOQ$bql_03 == "4"] <- 4
STUDOQ$bql_03_num[STUDOQ$bql_03 == "5"] <- 5
STUDOQ$bql_03_num[is.na(STUDOQ$bql_03)] <- NA

STUDOQ$bql_04_num[STUDOQ$bql_04 == "1"] <- 1
STUDOQ$bql_04_num[STUDOQ$bql_04 == "2"] <- 2
STUDOQ$bql_04_num[STUDOQ$bql_04 == "3"] <- 3
STUDOQ$bql_04_num[STUDOQ$bql_04 == "4"] <- 4
STUDOQ$bql_04_num[STUDOQ$bql_04 == "5"] <- 5
STUDOQ$bql_04_num[is.na(STUDOQ$bql_04)] <- NA

STUDOQ$bql_05_num[STUDOQ$bql_05 == "1"] <- 1
STUDOQ$bql_05_num[STUDOQ$bql_05 == "2"] <- 2
STUDOQ$bql_05_num[STUDOQ$bql_05 == "3"] <- 3
STUDOQ$bql_05_num[STUDOQ$bql_05 == "4"] <- 4
STUDOQ$bql_05_num[STUDOQ$bql_05 == "5"] <- 5
STUDOQ$bql_05_num[is.na(STUDOQ$bql_05)] <- NA

STUDOQ$bql_06_num[STUDOQ$bql_06 == "1"] <- 1
STUDOQ$bql_06_num[STUDOQ$bql_06 == "2"] <- 2
STUDOQ$bql_06_num[STUDOQ$bql_06 == "3"] <- 3
STUDOQ$bql_06_num[STUDOQ$bql_06 == "4"] <- 4
STUDOQ$bql_06_num[STUDOQ$bql_06 == "5"] <- 5
STUDOQ$bql_06_num[is.na(STUDOQ$bql_06)] <- NA

STUDOQ$bql_07_num[STUDOQ$bql_07 == "1"] <- 1
STUDOQ$bql_07_num[STUDOQ$bql_07 == "2"] <- 2
STUDOQ$bql_07_num[STUDOQ$bql_07 == "3"] <- 3
STUDOQ$bql_07_num[STUDOQ$bql_07 == "4"] <- 4
STUDOQ$bql_07_num[STUDOQ$bql_07 == "5"] <- 5
STUDOQ$bql_07_num[is.na(STUDOQ$bql_07)] <- NA

STUDOQ$bql_08_num[STUDOQ$bql_08 == "1"] <- 1
STUDOQ$bql_08_num[STUDOQ$bql_08 == "2"] <- 2
STUDOQ$bql_08_num[STUDOQ$bql_08 == "3"] <- 3
STUDOQ$bql_08_num[STUDOQ$bql_08 == "4"] <- 4
STUDOQ$bql_08_num[STUDOQ$bql_08 == "5"] <- 5
STUDOQ$bql_08_num[is.na(STUDOQ$bql_08)] <- NA

STUDOQ$bql_09_num[STUDOQ$bql_09 == "1"] <- 1
STUDOQ$bql_09_num[STUDOQ$bql_09 == "2"] <- 2
STUDOQ$bql_09_num[STUDOQ$bql_09 == "3"] <- 3
STUDOQ$bql_09_num[STUDOQ$bql_09 == "4"] <- 4
STUDOQ$bql_09_num[STUDOQ$bql_09 == "5"] <- 5
STUDOQ$bql_09_num[is.na(STUDOQ$bql_09)] <- NA

STUDOQ$bql_10_num[STUDOQ$bql_10 == "1"] <- 1
STUDOQ$bql_10_num[STUDOQ$bql_10 == "2"] <- 2
STUDOQ$bql_10_num[STUDOQ$bql_10 == "3"] <- 3
STUDOQ$bql_10_num[STUDOQ$bql_10 == "4"] <- 4
STUDOQ$bql_10_num[STUDOQ$bql_10 == "5"] <- 5
STUDOQ$bql_10_num[is.na(STUDOQ$bql_10)] <- NA

STUDOQ$bql_11_num[STUDOQ$bql_11 == "1"] <- 1
STUDOQ$bql_11_num[STUDOQ$bql_11 == "2"] <- 2
STUDOQ$bql_11_num[STUDOQ$bql_11 == "3"] <- 3
STUDOQ$bql_11_num[STUDOQ$bql_11 == "4"] <- 4
STUDOQ$bql_11_num[STUDOQ$bql_11 == "5"] <- 5
STUDOQ$bql_11_num[is.na(STUDOQ$bql_11)] <- NA

STUDOQ$bql_12_num[STUDOQ$bql_12 == "1"] <- 1
STUDOQ$bql_12_num[STUDOQ$bql_12 == "2"] <- 2
STUDOQ$bql_12_num[STUDOQ$bql_12 == "3"] <- 3
STUDOQ$bql_12_num[STUDOQ$bql_12 == "4"] <- 4
STUDOQ$bql_12_num[STUDOQ$bql_12 == "5"] <- 5
STUDOQ$bql_12_num[is.na(STUDOQ$bql_12)] <- NA

STUDOQ$bql_13_num[STUDOQ$bql_13 == "1"] <- 1
STUDOQ$bql_13_num[STUDOQ$bql_13 == "2"] <- 2
STUDOQ$bql_13_num[STUDOQ$bql_13 == "3"] <- 3
STUDOQ$bql_13_num[STUDOQ$bql_13 == "4"] <- 4
STUDOQ$bql_13_num[STUDOQ$bql_13 == "5"] <- 5
STUDOQ$bql_13_num[is.na(STUDOQ$bql_13)] <- NA

# Calculating BQL
STUDOQ$BQL <- ((STUDOQ$bql_01_num + STUDOQ$bql_02_num + STUDOQ$bql_03_num + STUDOQ$bql_04_num + STUDOQ$bql_05_num + STUDOQ$bql_06_num + STUDOQ$bql_07_num + STUDOQ$bql_08_num + STUDOQ$bql_09_num + STUDOQ$bql_10_num + STUDOQ$bql_11_num + STUDOQ$bql_12_num + STUDOQ$bql_13_num)/13)

summary(STUDOQ$BQL)
```

```
##    Min. 1st Qu.  Median    Mean 3rd Qu.    Max.    NA's 
##   1.231   3.692   4.192   4.059   4.615   5.000    4641
```

#### 3.2.5.3 Creation of number of comorbidities variable

```
# Transform all NA entries into true NAs

STUDOQ[] <- lapply(STUDOQ, function(x) {
    is.na(levels(x)) <- levels(x) == "NA"
    x
})

### Create number of baseline comorbidity variable
STUDOQ <- STUDOQ %>% 
          mutate(count_diabetes_2_baseline = ifelse(diabetes_2_baseline == "yes", 1,0)) %>% 
          mutate(count_hypertension_baseline = ifelse(hypertension_baseline == "yes", 1,0)) %>% 
          mutate(count_sleep_apnoea_baseline = ifelse(sleep_apnoea_baseline == "yes", 1,0)) %>% 
          mutate(count_dyslipidemia_baseline = ifelse(dyslipidemia_baseline == "yes", 1,0)) 
    
STUDOQ <- STUDOQ %>% 
          mutate(count_diabetes_2_baseline = as.numeric(count_diabetes_2_baseline)) %>% 
          mutate(count_hypertension_baseline = as.numeric(count_hypertension_baseline)) %>% 
          mutate(count_sleep_apnoea_baseline = as.numeric(count_sleep_apnoea_baseline)) %>% 
          mutate(count_dyslipidemia_baseline = as.numeric(count_dyslipidemia_baseline)) 

STUDOQ <- STUDOQ %>% 
          mutate(number_of_baseline_comorbidities = count_diabetes_2_baseline + count_hypertension_baseline +
                                                    count_sleep_apnoea_baseline +  count_dyslipidemia_baseline)  
      
STUDOQ <- STUDOQ %>% 
          mutate(number_of_baseline_comorbidities = ifelse(is.na(STUDOQ$diabetes_2_baseline) | is.na(hypertension_baseline) | is.na(sleep_apnoea_baseline) | is.na(dyslipidemia_baseline), NA, number_of_baseline_comorbidities   ))  %>% 
          mutate(number_of_baseline_comorbidities = as.factor(number_of_baseline_comorbidities)) %>% 
          mutate(number_of_baseline_comorbidities = fct_relevel(number_of_baseline_comorbidities, c("0", "1", "2", "3", "4")))


table(STUDOQ$number_of_baseline_comorbidities)
```

```
## 
##   0   1   2   3   4 
## 629 737 611 310  84
```

### 3.2.6 Tidy-up the data set

```
# Select and rearrange variables in the STUDOQ data set

STUDOQ <- select(STUDOQ, studygroup, sex, age_years, graduation, employment, optechnique, BMI, weight_baseline, weight_follow_up, total_weight_loss_percentage, total_weight_loss, BQL, fu1_completed, fu2_completed,  diabetes_2_baseline, diabetes_2_follow_up, hypertension_baseline, hypertension_follow_up, dyslipidemia_baseline, sleep_apnoea_baseline, number_of_baseline_comorbidities, pseudo_klinik_id )
                 
# Check out data set

str(STUDOQ)
```

```
## 'data.frame':    5859 obs. of  22 variables:
##  $ studygroup                      : Factor w/ 3 levels "No-COVID-19",..: 1 1 1 1 1 1 2 2 2 2 ...
##  $ sex                             : Factor w/ 2 levels "male","female": 2 2 1 2 1 2 2 1 2 2 ...
##  $ age_years                       : num  49 32 32 31 53 29 32 57 69 30 ...
##   ..- attr(*, "levels")= logi(0) 
##  $ graduation                      : Factor w/ 5 levels "unknown","low",..: NA NA NA NA NA 5 NA 2 NA NA ...
##  $ employment                      : Factor w/ 5 levels "unknown","not employed",..: NA NA NA NA NA 5 NA 5 NA NA ...
##  $ optechnique                     : Factor w/ 2 levels "Bypass","Sleeve": 1 2 2 2 2 2 1 2 2 1 ...
##  $ BMI                             : num  51 51 59 63 71 66 45 50 49 46 ...
##   ..- attr(*, "levels")= logi(0) 
##  $ weight_baseline                 : num  146 146 195 184 220 187 120 189 148 125 ...
##   ..- attr(*, "levels")= logi(0) 
##  $ weight_follow_up                : num  NA NA NA NA 138 140 89 172 NA 84 ...
##   ..- attr(*, "levels")= logi(0) 
##  $ total_weight_loss_percentage    : num  NA NA NA NA 37.3 ...
##   ..- attr(*, "levels")= logi(0) 
##  $ total_weight_loss               : num  NA NA NA NA 82 47 31 17 NA 41 ...
##   ..- attr(*, "levels")= logi(0) 
##  $ BQL                             : num  NA NA NA NA NA NA NA NA NA NA ...
##   ..- attr(*, "levels")= logi(0) 
##  $ fu1_completed                   : Factor w/ 2 levels "no","yes": 1 1 1 1 2 1 2 2 2 2 ...
##  $ fu2_completed                   : Factor w/ 2 levels "no","yes": 1 1 1 1 2 2 2 2 1 2 ...
##  $ diabetes_2_baseline             : Factor w/ 2 levels "yes","no": NA NA NA NA NA NA NA NA NA NA ...
##  $ diabetes_2_follow_up            : Factor w/ 2 levels "yes","no": NA NA NA NA 1 2 1 1 NA 2 ...
##  $ hypertension_baseline           : Factor w/ 2 levels "yes","no": NA NA NA NA NA NA NA NA NA NA ...
##  $ hypertension_follow_up          : Factor w/ 2 levels "yes","no": NA NA NA NA 1 2 1 1 NA 2 ...
##  $ dyslipidemia_baseline           : Factor w/ 2 levels "yes","no": NA NA NA NA NA NA NA NA NA NA ...
##  $ sleep_apnoea_baseline           : Factor w/ 2 levels "yes","no": NA NA NA NA NA NA NA NA NA NA ...
##  $ number_of_baseline_comorbidities: Factor w/ 5 levels "0","1","2","3",..: NA NA NA NA NA NA NA NA NA NA ...
##  $ pseudo_klinik_id                : chr  "33858" "33858" "33858" "70951" ...
##   ..- attr(*, "levels")= logi(0)
```

```
summary(STUDOQ)
```

```
##        studygroup       sex         age_years            graduation  
##  No-COVID-19:2664   male  :1715   Min.   :18.00   unknown     : 930  
##  COVID-19   :3195   female:4142   1st Qu.:34.00   low         :1149  
##  NP         :   0   NA's  :   2   Median :44.00   high        : 674  
##                                   Mean   :43.86   without     : 183  
##                                   3rd Qu.:53.00   intermediate:1480  
##                                   Max.   :81.00   NA's        :1443  
##                                                                      
##                    employment   optechnique        BMI         weight_baseline
##  unknown                : 579   Bypass:2199   Min.   : 15.00   Min.   : 50.0  
##  not employed           : 712   Sleeve:3660   1st Qu.: 43.00   1st Qu.:120.0  
##  retired                : 335                 Median : 48.00   Median :137.0  
##  housewife/ househusband: 337                 Mean   : 48.46   Mean   :140.3  
##  employed               :2458                 3rd Qu.: 53.00   3rd Qu.:156.0  
##  NA's                   :1438                 Max.   :101.00   Max.   :285.0  
##                                               NA's   :22       NA's   :22     
##  weight_follow_up total_weight_loss_percentage total_weight_loss
##  Min.   : 50.00   Min.   :-106.00              Min.   :-53.00   
##  1st Qu.: 80.00   1st Qu.:  25.33              1st Qu.: 33.00   
##  Median : 92.00   Median :  31.64              Median : 43.00   
##  Mean   : 95.61   Mean   :  31.20              Mean   : 43.81   
##  3rd Qu.:108.00   3rd Qu.:  37.67              3rd Qu.: 54.00   
##  Max.   :246.00   Max.   :  61.05              Max.   :126.00   
##  NA's   :1958     NA's   :1958                 NA's   :1958     
##       BQL        fu1_completed fu2_completed diabetes_2_baseline
##  Min.   :1.231   no  :1244     no  :1905     yes : 608          
##  1st Qu.:3.692   yes :4594     yes :3899     no  :1763          
##  Median :4.192   NA's:  21     NA's:  55     NA's:3488          
##  Mean   :4.059                                                  
##  3rd Qu.:4.615                                                  
##  Max.   :5.000                                                  
##  NA's   :4641                                                   
##  diabetes_2_follow_up hypertension_baseline hypertension_follow_up
##  yes : 706            yes :1363             yes :1558             
##  no  :3186            no  :1008             no  :2334             
##  NA's:1967            NA's:3488             NA's:1967             
##                                                                   
##                                                                   
##                                                                   
##                                                                   
##  dyslipidemia_baseline sleep_apnoea_baseline number_of_baseline_comorbidities
##  yes : 446             yes : 808             0   : 629                       
##  no  :1925             no  :1563             1   : 737                       
##  NA's:3488             NA's:3488             2   : 611                       
##                                              3   : 310                       
##                                              4   :  84                       
##                                              NA's:3488                       
##                                                                              
##  pseudo_klinik_id  
##  Length:5859       
##  Class :character  
##  Mode  :character  
##                    
##                    
##                    
##
```

```
# Get rid of NP level in studygroup variable
STUDOQ$studygroup <- droplevels(STUDOQ$studygroup, exclude = "NP")
```

## 3.3 Analysis of Missings

### 3.3.1 Missing Table

Missing Analysis: We calculate percentages of missing values for each
relevant variable for the total data set and stratified by study
group.

```
# Create dataframe to store the results

NA_table <- data.frame(variable = c("sex", "age", "graduation", "employment", "weight", "BMI", "OP technique", "diabetes type 2 at baseline", "hypertension at baseline", "dyslipidemia at baseline", "sleep_apnoea at baseline", "number of comorbidities at baseline",  "completed 3-month fu", "completed 1-year fu","% TWL", "BQL", "diabetes type 2 at fu", "hypertension at fu"), overall = c(rep(0, 18)), COVID_19 = c(rep(0, 18)), "No_COVID_19" = c(rep(0, 18)))

variable_list <- c("sex", "age_years", "graduation", "employment", "weight_baseline" , "BMI", "optechnique", "diabetes_2_baseline", "hypertension_baseline" , "dyslipidemia_baseline", "sleep_apnoea_baseline", "number_of_baseline_comorbidities", "fu1_completed", "fu2_completed", "total_weight_loss_percentage", "BQL", "diabetes_2_follow_up", "hypertension_follow_up")

# Define total number of observations

n <- nrow(STUDOQ)
n_COVID_19 <- nrow(STUDOQ[STUDOQ$studygroup == "COVID-19",])
n_No_COVID_19 <- nrow(STUDOQ[STUDOQ$studygroup == "No-COVID-19",]) 

# Fill the datframe for the overall group

for (i in 1:length(variable_list)) {
   
  missing_percentage <- sum(is.na(STUDOQ[[variable_list[i]]])) / n    
  
  NA_table[i, 2] <- missing_percentage 
  
}


# Fill the datframe for the COVID-19 group

STUDOQ_COVID_19_group <- filter(STUDOQ, studygroup == "COVID-19")
  
  
for (i in 1:length(variable_list)) {
   
  missing_percentage <- sum(is.na(STUDOQ_COVID_19_group[[variable_list[i]]])) / n_COVID_19    
  
  NA_table[i, 3] <- missing_percentage 
  
}


# Fill the datframe for the COVID-19 group

STUDOQ_No_COVID_19_group <- filter(STUDOQ, studygroup == "No-COVID-19")
  
  
for (i in 1:length(variable_list)) {
   
  missing_percentage <- sum(is.na(STUDOQ_No_COVID_19_group[[variable_list[i]]])) / n_No_COVID_19    
  
  NA_table[i, 4] <- missing_percentage 
  
}

# Check out the table

NA_table
```

```
##                               variable      overall    COVID_19  No_COVID_19
## 1                                  sex 0.0003413552 0.000312989 0.0003753754
## 2                                  age 0.0000000000 0.000000000 0.0000000000
## 3                           graduation 0.2462877624 0.237871674 0.2563813814
## 4                           employment 0.2454343745 0.236619718 0.2560060060
## 5                               weight 0.0037549070 0.000000000 0.0082582583
## 6                                  BMI 0.0037549070 0.000000000 0.0082582583
## 7                         OP technique 0.0000000000 0.000000000 0.0000000000
## 8          diabetes type 2 at baseline 0.5953234340 0.619405321 0.5664414414
## 9             hypertension at baseline 0.5953234340 0.619405321 0.5664414414
## 10            dyslipidemia at baseline 0.5953234340 0.619405321 0.5664414414
## 11            sleep_apnoea at baseline 0.5953234340 0.619405321 0.5664414414
## 12 number of comorbidities at baseline 0.5953234340 0.619405321 0.5664414414
## 13                completed 3-month fu 0.0035842294 0.005320814 0.0015015015
## 14                 completed 1-year fu 0.0093872675 0.012832551 0.0052552553
## 15                               % TWL 0.3341867213 0.371205008 0.2897897898
## 16                                 BQL 0.7921146953 0.801877934 0.7804054054
## 17               diabetes type 2 at fu 0.3357228196 0.370892019 0.2935435435
## 18                  hypertension at fu 0.3357228196 0.370892019 0.2935435435
```

When we compare the missing shares for the baseline characteristics
between the two studygroups, we see only slight differences. When we
look at the outcomes there are larger differences. However, they are due
to the fact that in the COVID-19 group less patients participated in the
1 year follow up. Hence, these differences in missings make absolute
sense.

### 3.3.2 Handling of Missings

We handle missings in the following way:

- Descriptive statistics are always only calculated for those
  patients where information for the respective variable is not
  missing.
- In case of the regression models all observations are dismissed
  from the analysis that have a missing in the respective outcome variable
  or in one of the numeric baseline characteristics (age or BMI). For the
  categorical variables, the NA`s are either added to an already existing
  category (e.g. “unknown” category of employment/graduation) or an
  additional NA category is created (e.g. number of
  comorbidities).

```
### Create baseline characteristic variables for the regression models

# Employment

summary(STUDOQ$employment)
```

```
##                 unknown            not employed                 retired 
##                     579                     712                     335 
## housewife/ househusband                employed                    NA's 
##                     337                    2458                    1438
```

```
STUDOQ <- STUDOQ %>% mutate(employment_reg = employment)

STUDOQ$employment_reg[is.na(STUDOQ$employment)] <- "unknown"

summary(STUDOQ$employment_reg)
```

```
##                 unknown            not employed                 retired 
##                    2017                     712                     335 
## housewife/ househusband                employed 
##                     337                    2458
```

```
STUDOQ <- STUDOQ %>% mutate(employment_reg = fct_relevel(employment_reg, c("employed", "housewife/ househusband", "retired", "not employed", "unknown")))

summary(STUDOQ$employment)
```

```
##                 unknown            not employed                 retired 
##                     579                     712                     335 
## housewife/ househusband                employed                    NA's 
##                     337                    2458                    1438
```

```
# Graduation

summary(STUDOQ$graduation)
```

```
##      unknown          low         high      without intermediate         NA's 
##          930         1149          674          183         1480         1443
```

```
STUDOQ <- STUDOQ %>% mutate(graduation_reg = graduation)

STUDOQ$graduation_reg[is.na(STUDOQ$graduation)] <- "unknown"

STUDOQ <- STUDOQ %>% mutate(graduation_reg = fct_relevel(graduation_reg, c("high", "intermediate", "low", "without", "unknown")))

summary(STUDOQ$graduation_reg)
```

```
##         high intermediate          low      without      unknown 
##          674         1480         1149          183         2373
```

```
# Sex

summary(STUDOQ$sex)
```

```
##   male female   NA's 
##   1715   4142      2
```

```
STUDOQ <- STUDOQ %>% mutate(sex_reg = sex)

STUDOQ$sex_reg[is.na(STUDOQ$sex)] <- "female"

STUDOQ <- STUDOQ %>% mutate(sex_reg = fct_relevel(sex_reg, c("female", "male")))

summary(STUDOQ$sex_reg)
```

```
## female   male 
##   4144   1715
```

```
# Number of comorbidities

STUDOQ <- STUDOQ %>% mutate(number_of_baseline_comorbidities_reg = number_of_baseline_comorbidities)

STUDOQ$number_of_baseline_comorbidities_reg[is.na(STUDOQ$number_of_baseline_comorbidities)] <- "NA"

STUDOQ <- STUDOQ %>% 
  mutate(number_of_baseline_comorbidities_reg = ifelse(is.na(number_of_baseline_comorbidities_reg), "NA", number_of_baseline_comorbidities_reg)) %>%   
  mutate(number_of_baseline_comorbidities_reg = as.factor(number_of_baseline_comorbidities_reg)) %>% 
  mutate(number_of_baseline_comorbidities_reg = fct_recode(number_of_baseline_comorbidities_reg, "0" = "1", "1" = "2", "2" ="3", "3" = "4", "4" = "5", "NA" = "NA"))

summary(STUDOQ$number_of_baseline_comorbidities_reg)
```

```
##    0    1    2    3    4   NA 
##  629  737  611  310   84 3488
```

## 3.4 Last Steps

### 3.4.1 Relevel binary outcomes for regression analysis

```
# fu1_completed
STUDOQ$fu1_completed <- relevel(STUDOQ$fu1_completed, ref = "no")

# fu2_completed
STUDOQ$fu2_completed <- relevel(STUDOQ$fu2_completed, ref = "no")

# diabetes_2_follow_up
STUDOQ$diabetes_2_follow_up <- relevel(STUDOQ$diabetes_2_follow_up, ref = "no")

# hypertension_follow_up
STUDOQ$hypertension_follow_up <- relevel(STUDOQ$hypertension_follow_up, ref = "no")
```

### 3.4.2 Create final data set

```
# Create the final dataset that is used in the analysis section

STUDOQ_final <- STUDOQ

str(STUDOQ_final)
```

```
## 'data.frame':    5859 obs. of  26 variables:
##  $ studygroup                          : Factor w/ 2 levels "No-COVID-19",..: 1 1 1 1 1 1 2 2 2 2 ...
##  $ sex                                 : Factor w/ 2 levels "male","female": 2 2 1 2 1 2 2 1 2 2 ...
##  $ age_years                           : num  49 32 32 31 53 29 32 57 69 30 ...
##   ..- attr(*, "levels")= logi(0) 
##  $ graduation                          : Factor w/ 5 levels "unknown","low",..: NA NA NA NA NA 5 NA 2 NA NA ...
##  $ employment                          : Factor w/ 5 levels "unknown","not employed",..: NA NA NA NA NA 5 NA 5 NA NA ...
##  $ optechnique                         : Factor w/ 2 levels "Bypass","Sleeve": 1 2 2 2 2 2 1 2 2 1 ...
##  $ BMI                                 : num  51 51 59 63 71 66 45 50 49 46 ...
##   ..- attr(*, "levels")= logi(0) 
##  $ weight_baseline                     : num  146 146 195 184 220 187 120 189 148 125 ...
##   ..- attr(*, "levels")= logi(0) 
##  $ weight_follow_up                    : num  NA NA NA NA 138 140 89 172 NA 84 ...
##   ..- attr(*, "levels")= logi(0) 
##  $ total_weight_loss_percentage        : num  NA NA NA NA 37.3 ...
##   ..- attr(*, "levels")= logi(0) 
##  $ total_weight_loss                   : num  NA NA NA NA 82 47 31 17 NA 41 ...
##   ..- attr(*, "levels")= logi(0) 
##  $ BQL                                 : num  NA NA NA NA NA NA NA NA NA NA ...
##   ..- attr(*, "levels")= logi(0) 
##  $ fu1_completed                       : Factor w/ 2 levels "no","yes": 1 1 1 1 2 1 2 2 2 2 ...
##  $ fu2_completed                       : Factor w/ 2 levels "no","yes": 1 1 1 1 2 2 2 2 1 2 ...
##  $ diabetes_2_baseline                 : Factor w/ 2 levels "yes","no": NA NA NA NA NA NA NA NA NA NA ...
##  $ diabetes_2_follow_up                : Factor w/ 2 levels "no","yes": NA NA NA NA 2 1 2 2 NA 1 ...
##  $ hypertension_baseline               : Factor w/ 2 levels "yes","no": NA NA NA NA NA NA NA NA NA NA ...
##  $ hypertension_follow_up              : Factor w/ 2 levels "no","yes": NA NA NA NA 2 1 2 2 NA 1 ...
##  $ dyslipidemia_baseline               : Factor w/ 2 levels "yes","no": NA NA NA NA NA NA NA NA NA NA ...
##  $ sleep_apnoea_baseline               : Factor w/ 2 levels "yes","no": NA NA NA NA NA NA NA NA NA NA ...
##  $ number_of_baseline_comorbidities    : Factor w/ 5 levels "0","1","2","3",..: NA NA NA NA NA NA NA NA NA NA ...
##  $ pseudo_klinik_id                    : chr  "33858" "33858" "33858" "70951" ...
##   ..- attr(*, "levels")= logi(0) 
##  $ employment_reg                      : Factor w/ 5 levels "employed","housewife/ househusband",..: 5 5 5 5 5 1 5 1 5 5 ...
##  $ graduation_reg                      : Factor w/ 5 levels "high","intermediate",..: 5 5 5 5 5 2 5 3 5 5 ...
##  $ sex_reg                             : Factor w/ 2 levels "female","male": 1 1 2 1 2 1 1 2 1 1 ...
##  $ number_of_baseline_comorbidities_reg: Factor w/ 6 levels "0","1","2","3",..: 6 6 6 6 6 6 6 6 6 6 ...
```

```
save(STUDOQ_final, file = "STUDOQ_final")

load("STUDOQ_final")
```

# 4 Data Analysis

After preparing the dataset, we will conduct the analyses that are
needed to answer our research questions in the section below. We will
create tables that include all the information that are needed to create
the tables for the paper and the appendix.

## 4.1 Descriptive Analysis

### 4.1.1 Numbers needed for the text

All numbers that are not included in tables but in the text of the
paper are derived below.

```
# Create dataframe to store the results

table_text_numbers <- data.frame(variable = c("n_total", "n_NO_COVID_19_group", "n_COVID_19_group", "age_min", "age_max", "center_number"), 
                                 value = c(rep(0, 6)))

# Calculate group sizes

n_total <- nrow(STUDOQ_final)
n_NO_COVID_19_group <- nrow(STUDOQ_final[STUDOQ_final$studygroup == "No-COVID-19",])
n_COVID_19_group <- nrow(STUDOQ_final[STUDOQ_final$studygroup == "COVID-19",])
age_min <- min(STUDOQ_final$age_years) 
age_max <- max(STUDOQ_final$age_years)
center_number <- length(unique(STUDOQ_final$pseudo_klinik_id))

# Fill the dataframe for the overall group

table_text_numbers[1, 2] <- n_total
table_text_numbers[2, 2] <- n_NO_COVID_19_group
table_text_numbers[3, 2] <- n_COVID_19_group
table_text_numbers[4, 2] <- age_min
table_text_numbers[5, 2] <- age_max
table_text_numbers[6, 2] <- center_number

# Check out the table

table_text_numbers
```

```
##              variable value
## 1             n_total  5859
## 2 n_NO_COVID_19_group  2664
## 3    n_COVID_19_group  3195
## 4             age_min    18
## 5             age_max    81
## 6       center_number   154
```

```
# Create Excel Table that serves as Input file for creating Table A1 of the paper

write.xlsx(table_text_numbers, "numbers_for_text_input.xlsx")
```

### 4.1.2 Table 1

**Comparison of study groups**: **Table1**
package was used to create a table to compare studygroups with respect
to their baseline characteristics.

Because of the missing data, each outcome is based on a different
study population.

```
### Categorical variables

#  For the comparison table, the desired order of the categories from the covariate variables was determined.
desired_order_sex <- c("female", "male")
desired_order_graduation <- c("high", "intermediate", "low", "without", "unknown")
desired_order_employment <- c("employed", "housewife/ househusband", "retired", "not employed", "unknown")
desired_order_T2D_baseline <- c("yes", "no")
desired_order_dyslipidemia_baseline <- c("yes", "no")
desired_order_sleep_apnoea_baseline <- c("yes", "no")
desired_order_hypertension_baseline <- c("yes", "no")
#
STUDOQ_final$sex  <- factor(STUDOQ_final$sex, levels = desired_order_sex)
STUDOQ_final$graduation  <- factor(STUDOQ_final$graduation, levels = desired_order_graduation)
STUDOQ_final$employment <- factor(STUDOQ_final$employment, levels = desired_order_employment)
STUDOQ_final$diabetes_2_baseline  <- factor(STUDOQ_final$diabetes_2_baseline, levels = desired_order_T2D_baseline)
STUDOQ_final$hypertension_baseline  <- factor(STUDOQ_final$hypertension_baseline, levels = desired_order_hypertension_baseline)
STUDOQ_final$dyslipidemia_baseline  <- factor(STUDOQ_final$dyslipidemia_baseline, levels = desired_order_dyslipidemia_baseline)
STUDOQ_final$sleep_apnoea_baseline  <- factor(STUDOQ_final$sleep_apnoea_baseline, levels = desired_order_sleep_apnoea_baseline)

# Create table

table_1_cat <- table1(~ sex + graduation + employment + optechnique + diabetes_2_baseline + hypertension_baseline +  dyslipidemia_baseline + sleep_apnoea_baseline | studygroup, data=STUDOQ_final, overall = c(left="Overall"), render.missing=NULL, render.categorical="PCTnoNA%")

table_1_cat <- as.data.frame(table_1_cat)


names(table_1_cat)[1] <- "Variable"
names(table_1_cat)[3] <- "No_COVID_19"
names(table_1_cat)[4] <- "COVID_19"

### Numeric Variables

# Create df to store the data 
table_1_num <- data.frame(Variable = c("age (mean)", "age (SD)",  "weight (mean)",  "weight (SD)", "BMI (mean)", "BMI (SD)"),
                          Overall = c(rep(0, 6)), 
                          No_COVID_19 = c(rep(0, 6)), 
                          COVID_19 = c(rep(0, 6)))

# Fill the data frame with mean and sd information for the numerical variables

# Create studygroup subsamples

STUDOQ_final_NO_COVID_19 <- filter(STUDOQ_final, studygroup =="No-COVID-19")

STUDOQ_final_COVID_19 <- filter(STUDOQ_final, studygroup =="COVID-19")


## age
# mean
table_1_num[1,2] <- round(mean(STUDOQ_final$age_years, na.rm = TRUE), digits = 1)
table_1_num[1,3] <- round(mean(STUDOQ_final_NO_COVID_19$age_years, na.rm = TRUE), digits = 1)
table_1_num[1,4] <- round(mean(STUDOQ_final_COVID_19$age_years, na.rm = TRUE), digits = 1)

# sd 
table_1_num[2,2] <- round(sd(STUDOQ_final$age_years, na.rm = TRUE), digits = 1)
table_1_num[2,3] <- round(sd(STUDOQ_final_NO_COVID_19$age_years, na.rm = TRUE), digits = 1)
table_1_num[2,4] <- round(sd(STUDOQ_final_COVID_19$age_years, na.rm = TRUE), digits = 1)

## weight
# mean
table_1_num[3,2] <- round(mean(STUDOQ_final$weight_baseline, na.rm = TRUE), digits = 1)
table_1_num[3,3] <- round(mean(STUDOQ_final_NO_COVID_19$weight_baseline, na.rm = TRUE), digits = 1)
table_1_num[3,4] <- round(mean(STUDOQ_final_COVID_19$weight_baseline, na.rm = TRUE), digits = 1)


# sd 
table_1_num[4,2] <- round(sd(STUDOQ_final$weight_baseline, na.rm = TRUE), digits = 1)
table_1_num[4,3] <- round(sd(STUDOQ_final_NO_COVID_19$weight_baseline, na.rm = TRUE), digits = 1)
table_1_num[4,4] <- round(sd(STUDOQ_final_COVID_19$weight_baseline, na.rm = TRUE), digits = 1)

## BMI
# mean
table_1_num[5,2] <- round(mean(STUDOQ_final$BMI, na.rm = TRUE), digits = 1)
table_1_num[5,3] <- round(mean(STUDOQ_final_NO_COVID_19$BMI, na.rm = TRUE), digits = 1)
table_1_num[5,4] <- round(mean(STUDOQ_final_COVID_19$BMI, na.rm = TRUE), digits = 1)

# sd 
table_1_num[6,2] <- round(sd(STUDOQ_final$BMI, na.rm = TRUE), digits = 1)
table_1_num[6,3] <- round(sd(STUDOQ_final_NO_COVID_19$BMI, na.rm = TRUE), digits = 1)
table_1_num[6,4] <- round(sd(STUDOQ_final_COVID_19$BMI, na.rm = TRUE), digits = 1)


### Combine numeric with categorical variables
table_1 <- rbind(table_1_cat, table_1_num)

### Check out the table
table_1
```

```
##                     Variable  Overall No_COVID_19 COVID_19
## 1                            (N=5859)    (N=2664) (N=3195)
## 2                        sex                              
## 3                     female    70.7%       71.1%    70.4%
## 4                       male    29.3%       28.9%    29.6%
## 5                 graduation                              
## 6                       high    15.3%       14.6%    15.8%
## 7               intermediate    33.5%       34.3%    32.9%
## 8                        low    26.0%       26.8%    25.4%
## 9                    without     4.1%        4.4%     3.9%
## 10                   unknown    21.1%       19.9%    22.0%
## 11                employment                              
## 12                  employed    55.6%       54.0%    56.9%
## 13   housewife/ househusband     7.6%        8.0%     7.3%
## 14                   retired     7.6%        8.0%     7.2%
## 15              not employed    16.1%       16.9%    15.5%
## 16                   unknown    13.1%       13.1%    13.1%
## 17               optechnique                              
## 18                    Bypass    37.5%       40.3%    35.2%
## 19                    Sleeve    62.5%       59.7%    64.8%
## 20       diabetes_2_baseline                              
## 21                       yes    25.6%       26.0%    25.3%
## 22                        no    74.4%       74.0%    74.7%
## 23     hypertension_baseline                              
## 24                       yes    57.5%       58.3%    56.7%
## 25                        no    42.5%       41.7%    43.3%
## 26     dyslipidemia_baseline                              
## 27                       yes    18.8%       18.4%    19.2%
## 28                        no    81.2%       81.6%    80.8%
## 29     sleep_apnoea_baseline                              
## 30                       yes    34.1%       34.4%    33.8%
## 31                        no    65.9%       65.6%    66.2%
## 32                age (mean)     43.9        44.1     43.7
## 33                  age (SD)     11.8        11.8     11.8
## 34             weight (mean)    140.3         141    139.7
## 35               weight (SD)     27.4        27.7     27.1
## 36                BMI (mean)     48.5        48.6     48.4
## 37                  BMI (SD)      7.8         7.8      7.7
```

```
### Output input table
write.xlsx(table_1, "Table_1_R_input.xlsx")
```

### 4.1.3 Table 2

In a next step, we create a table that allows us to compare the
outcomes between our two studygroups.

```
### Set up dataframe to store results

table_2 <- data.frame(    outcome = c("%TWL", "BQL", "3-month fu participation", "1-year fu participation", "Type-2 Diabetes", "Hypertension"),
                          N = c(rep(0, 6)),
                          No_COVID_19 = c(rep(0, 6)), 
                          No_COVID_19_SD = c(rep(0, 6)),
                          COVID_19 = c(rep(0, 6)),
                          COVID_19_SD = c(rep(0, 6)),
                          p_value = c(rep(0, 6)))

### Calculate and store results

## %TWL

table_2[1,2] <- nrow(filter(STUDOQ_final, !is.na(total_weight_loss_percentage)))
table_2[1,3] <- mean(STUDOQ_final$total_weight_loss_percentage[STUDOQ_final$studygroup == "No-COVID-19"], na.rm = TRUE) 
table_2[1,4] <- sd(STUDOQ_final$total_weight_loss_percentage[STUDOQ_final$studygroup == "No-COVID-19"], na.rm = TRUE) 
table_2[1,5] <- mean(STUDOQ_final$total_weight_loss_percentage[STUDOQ_final$studygroup == "COVID-19"], na.rm = TRUE) 
table_2[1,6] <- sd(STUDOQ_final$total_weight_loss_percentage[STUDOQ_final$studygroup == "COVID-19"], na.rm = TRUE) 
table_2[1,7] <- t.test(STUDOQ_final$total_weight_loss_percentage[STUDOQ_final$studygroup == "COVID-19"], STUDOQ_final$total_weight_loss_percentage[STUDOQ_final$studygroup == "No-COVID-19"])$p.value

 
## BQL

table_2[2,2] <- nrow(filter(STUDOQ_final, !is.na(BQL)))
table_2[2,3] <- mean(STUDOQ_final$BQL[STUDOQ_final$studygroup == "No-COVID-19"], na.rm = TRUE) 
table_2[2,4] <- sd(STUDOQ_final$BQL[STUDOQ_final$studygroup == "No-COVID-19"], na.rm = TRUE) 
table_2[2,5] <- mean(STUDOQ_final$BQL[STUDOQ_final$studygroup == "COVID-19"], na.rm = TRUE) 
table_2[2,6] <- sd(STUDOQ_final$BQL[STUDOQ_final$studygroup == "COVID-19"], na.rm = TRUE) 
table_2[2,7] <- t.test(STUDOQ_final$BQL[STUDOQ_final$studygroup == "COVID-19"], STUDOQ_final$BQL[STUDOQ_final$studygroup == "No-COVID-19"])$p.value


## 3-month fu participation

table_2[3,2] <- nrow(filter(STUDOQ_final, !is.na(fu1_completed)))
table_2[3,3] <- nrow(filter(STUDOQ_final, fu1_completed == "yes" & studygroup == "No-COVID-19"))/nrow(filter(STUDOQ_final, fu1_completed %in% c("yes", "no") & studygroup == "No-COVID-19")) 


table_2[3,4] <- NA
table_2[3,5] <- nrow(filter(STUDOQ_final, fu1_completed == "yes" & studygroup == "COVID-19"))/nrow(filter(STUDOQ_final, fu1_completed %in% c("yes", "no") & studygroup == "COVID-19")) 

table_2[3,6] <- NA 
table_2[3,7] <- chisq.test(STUDOQ_final$fu1_completed, STUDOQ_final$studygroup)$p.value

## 1-year fu participation


table_2[4,2] <- nrow(filter(STUDOQ_final, !is.na(fu2_completed)))
table_2[4,3] <- nrow(filter(STUDOQ_final, fu2_completed == "yes" & studygroup == "No-COVID-19"))/nrow(filter(STUDOQ_final, fu2_completed %in% c("yes", "no") & studygroup == "No-COVID-19")) 

table_2[4,4] <- NA
table_2[4,5] <- nrow(filter(STUDOQ_final, fu2_completed == "yes" & studygroup == "COVID-19"))/nrow(filter(STUDOQ_final, fu2_completed %in% c("yes", "no") & studygroup == "COVID-19")) 

table_2[4,6] <- NA 
table_2[4,7] <- chisq.test(STUDOQ_final$fu2_completed, STUDOQ_final$studygroup)$p.value

## Type-2 Diabetes


table_2[5,2] <- nrow(filter(STUDOQ_final, !is.na(diabetes_2_follow_up)))
table_2[5,3] <- nrow(filter(STUDOQ_final, diabetes_2_follow_up == "yes" & studygroup == "No-COVID-19"))/nrow(filter(STUDOQ_final, diabetes_2_follow_up %in% c("yes", "no") & studygroup == "No-COVID-19")) 


table_2[5,4] <- NA
table_2[5,5] <- nrow(filter(STUDOQ_final, diabetes_2_follow_up == "yes" & studygroup == "COVID-19"))/nrow(filter(STUDOQ_final, diabetes_2_follow_up %in% c("yes", "no") & studygroup == "COVID-19")) 
table_2[5,6] <- NA 
table_2[5,7] <- chisq.test(STUDOQ_final$diabetes_2_follow_up, STUDOQ_final$studygroup)$p.value

## Hypertension

table_2[6,2] <- nrow(filter(STUDOQ_final, !is.na(hypertension_follow_up)))
table_2[6,3] <- nrow(filter(STUDOQ_final, hypertension_follow_up == "yes" & studygroup == "No-COVID-19"))/nrow(filter(STUDOQ_final, hypertension_follow_up %in% c("yes", "no") & studygroup == "No-COVID-19")) 


table_2[6,4] <- NA
table_2[6,5] <- nrow(filter(STUDOQ_final, hypertension_follow_up == "yes" & studygroup == "COVID-19"))/nrow(filter(STUDOQ_final, hypertension_follow_up %in% c("yes", "no") & studygroup == "COVID-19")) 

table_2[6,6] <- NA 
table_2[6,7] <- chisq.test(STUDOQ_final$hypertension_follow_up, STUDOQ_final$studygroup)$p.value


## Check out the result

table_2
```

```
##                    outcome    N No_COVID_19 No_COVID_19_SD   COVID_19
## 1                     %TWL 3901  31.0957912     10.1384785 31.2995001
## 2                      BQL 1218   4.1153189      0.6542509  4.0060761
## 3 3-month fu participation 5838   0.8274436             NA  0.7529893
## 4  1-year fu participation 5804   0.7132075             NA  0.6369689
## 5          Type-2 Diabetes 3892   0.1849097             NA  0.1781095
## 6             Hypertension 3892   0.4160468             NA  0.3855721
##   COVID_19_SD              p_value
## 1  10.1320512 0.530444672543864737
## 2   0.7271734 0.005873667977156982
## 3          NA 0.000000000005707635
## 4          NA 0.000000000861373648
## 5          NA 0.611071784052876987
## 6          NA 0.056603671089758931
```

```
# Do some roundig

table_2 <- table_2 %>% 
            mutate(No_COVID_19 = round(No_COVID_19, digits = 3)) %>% 
            mutate(No_COVID_19_SD = round(No_COVID_19_SD, digits = 2)) %>% 
            mutate(COVID_19 = round(COVID_19, digits = 3)) %>% 
            mutate(COVID_19_SD = round(COVID_19_SD, digits = 2)) %>%
            mutate(p_value = round(p_value, digits = 3)) 
  
### Output input table
write.xlsx(table_2, "Table_2_R_input.xlsx")
```

## 4.2 Regression Analysis

**Multivariable regression models** were used to answer
the research questions.

- For the primary outcomes %TWL and BQL, linear regression models are
  used.
- Logistic regression models are applied for the analysis of the
  secondary outcomes and reported as Odds Ratios.
- To control for potential observed confounders, the above-mentioned
  co-variables are included in the regression models.

### 4.2.1 Table 3

```
#############################################################################
######## Estimate regression models and check out results ###################
#############################################################################

#####################
##### %TWL ##########
#####################


model_1 <- lm(total_weight_loss_percentage ~ studygroup + age_years + BMI + optechnique  + sex_reg + graduation_reg + 
                                             employment_reg + number_of_baseline_comorbidities_reg, 
                                             data = STUDOQ_final)

summary(model_1)
```

```
## 
## Call:
## lm(formula = total_weight_loss_percentage ~ studygroup + age_years + 
##     BMI + optechnique + sex_reg + graduation_reg + employment_reg + 
##     number_of_baseline_comorbidities_reg, data = STUDOQ_final)
## 
## Residuals:
##      Min       1Q   Median       3Q      Max 
## -123.309   -5.335    0.394    5.972   32.463 
## 
## Coefficients:
##                                         Estimate Std. Error t value
## (Intercept)                            32.261192   1.311510  24.599
## studygroupCOVID-19                      0.286824   0.308529   0.930
## age_years                              -0.177726   0.014658 -12.125
## BMI                                     0.215637   0.020980  10.278
## optechniqueSleeve                      -2.761320   0.326051  -8.469
## sex_regmale                            -1.019358   0.359063  -2.839
## graduation_regintermediate              0.007697   0.541289   0.014
## graduation_reglow                       0.409310   0.573517   0.714
## graduation_regwithout                  -1.744753   1.023217  -1.705
## graduation_regunknown                  -0.361175   0.624165  -0.579
## employment_reghousewife/ househusband  -1.107884   0.694861  -1.594
## employment_regretired                  -2.319223   0.682808  -3.397
## employment_regnot employed             -0.764361   0.517768  -1.476
## employment_regunknown                  -0.148982   0.515839  -0.289
## number_of_baseline_comorbidities_reg1   0.107228   0.622754   0.172
## number_of_baseline_comorbidities_reg2  -1.537500   0.666955  -2.305
## number_of_baseline_comorbidities_reg3  -2.619200   0.821200  -3.189
## number_of_baseline_comorbidities_reg4  -2.456230   1.329444  -1.848
## number_of_baseline_comorbidities_regNA -1.786333   0.530542  -3.367
##                                                    Pr(>|t|)    
## (Intercept)                            < 0.0000000000000002 ***
## studygroupCOVID-19                                 0.352610    
## age_years                              < 0.0000000000000002 ***
## BMI                                    < 0.0000000000000002 ***
## optechniqueSleeve                      < 0.0000000000000002 ***
## sex_regmale                                        0.004550 ** 
## graduation_regintermediate                         0.988655    
## graduation_reglow                                  0.475466    
## graduation_regwithout                              0.088244 .  
## graduation_regunknown                              0.562857    
## employment_reghousewife/ househusband              0.110929    
## employment_regretired                              0.000689 ***
## employment_regnot employed                         0.139955    
## employment_regunknown                              0.772739    
## number_of_baseline_comorbidities_reg1              0.863302    
## number_of_baseline_comorbidities_reg2              0.021205 *  
## number_of_baseline_comorbidities_reg3              0.001437 ** 
## number_of_baseline_comorbidities_reg4              0.064742 .  
## number_of_baseline_comorbidities_regNA             0.000767 ***
## ---
## Signif. codes:  0 '***' 0.001 '**' 0.01 '*' 0.05 '.' 0.1 ' ' 1
## 
## Residual standard error: 9.572 on 3882 degrees of freedom
##   (1958 Beobachtungen als fehlend gelöscht)
## Multiple R-squared:  0.1119, Adjusted R-squared:  0.1078 
## F-statistic: 27.19 on 18 and 3882 DF,  p-value: < 0.00000000000000022
```

```
####################
##### BQL ##########
####################

model_2 <- lm(BQL ~ studygroup + age_years + BMI + optechnique  + sex_reg + graduation_reg + 
                    employment_reg + number_of_baseline_comorbidities_reg, 
                    data = STUDOQ_final)
              
summary(model_2)
```

```
## 
## Call:
## lm(formula = BQL ~ studygroup + age_years + BMI + optechnique + 
##     sex_reg + graduation_reg + employment_reg + number_of_baseline_comorbidities_reg, 
##     data = STUDOQ_final)
## 
## Residuals:
##     Min      1Q  Median      3Q     Max 
## -2.7702 -0.3715  0.1042  0.4660  1.4445 
## 
## Coefficients:
##                                         Estimate Std. Error t value
## (Intercept)                             5.322620   0.159620  33.345
## studygroupCOVID-19                     -0.140420   0.037686  -3.726
## age_years                              -0.004732   0.001860  -2.545
## BMI                                    -0.015696   0.002672  -5.874
## optechniqueSleeve                      -0.150580   0.038966  -3.864
## sex_regmale                             0.071538   0.043689   1.637
## graduation_regintermediate             -0.040365   0.052264  -0.772
## graduation_reglow                      -0.122278   0.055907  -2.187
## graduation_regwithout                  -0.304240   0.107798  -2.822
## graduation_regunknown                  -0.160506   0.096803  -1.658
## employment_reghousewife/ househusband  -0.164830   0.071049  -2.320
## employment_regretired                  -0.416746   0.073361  -5.681
## employment_regnot employed             -0.354061   0.054151  -6.538
## employment_regunknown                  -0.260681   0.087721  -2.972
## number_of_baseline_comorbidities_reg1   0.038281   0.061608   0.621
## number_of_baseline_comorbidities_reg2   0.031778   0.068253   0.466
## number_of_baseline_comorbidities_reg3  -0.085794   0.085349  -1.005
## number_of_baseline_comorbidities_reg4  -0.187512   0.142266  -1.318
## number_of_baseline_comorbidities_regNA  0.078387   0.056029   1.399
##                                                    Pr(>|t|)    
## (Intercept)                            < 0.0000000000000002 ***
## studygroupCOVID-19                                 0.000204 ***
## age_years                                          0.011067 *  
## BMI                                         0.0000000055126 ***
## optechniqueSleeve                                  0.000117 ***
## sex_regmale                                        0.101805    
## graduation_regintermediate                         0.440065    
## graduation_reglow                                  0.028923 *  
## graduation_regwithout                              0.004846 ** 
## graduation_regunknown                              0.097567 .  
## employment_reghousewife/ househusband              0.020511 *  
## employment_regretired                       0.0000000168160 ***
## employment_regnot employed                  0.0000000000918 ***
## employment_regunknown                              0.003020 ** 
## number_of_baseline_comorbidities_reg1              0.534481    
## number_of_baseline_comorbidities_reg2              0.641594    
## number_of_baseline_comorbidities_reg3              0.314992    
## number_of_baseline_comorbidities_reg4              0.187744    
## number_of_baseline_comorbidities_regNA             0.162060    
## ---
## Signif. codes:  0 '***' 0.001 '**' 0.01 '*' 0.05 '.' 0.1 ' ' 1
## 
## Residual standard error: 0.644 on 1199 degrees of freedom
##   (4641 Beobachtungen als fehlend gelöscht)
## Multiple R-squared:  0.1541, Adjusted R-squared:  0.1414 
## F-statistic: 12.14 on 18 and 1199 DF,  p-value: < 0.00000000000000022
```

```
############################################################
##### Participation 3-month follow-up appointment ##########
############################################################

model_3 <- glm(fu1_completed ~ studygroup + age_years + BMI + optechnique  + sex_reg + graduation_reg + 
                    employment_reg + number_of_baseline_comorbidities_reg,
                    family = binomial,
                    data = STUDOQ_final)
              
summary(model_3)
```

```
## 
## Call:
## glm(formula = fu1_completed ~ studygroup + age_years + BMI + 
##     optechnique + sex_reg + graduation_reg + employment_reg + 
##     number_of_baseline_comorbidities_reg, family = binomial, 
##     data = STUDOQ_final)
## 
## Coefficients:
##                                         Estimate Std. Error z value
## (Intercept)                             1.551901   0.279578   5.551
## studygroupCOVID-19                     -0.483198   0.067925  -7.114
## age_years                               0.009671   0.003023   3.199
## BMI                                     0.002020   0.004399   0.459
## optechniqueSleeve                      -0.274710   0.072821  -3.772
## sex_regmale                            -0.286583   0.073901  -3.878
## graduation_regintermediate              0.228529   0.127205   1.797
## graduation_reglow                       0.167766   0.133909   1.253
## graduation_regwithout                  -0.416918   0.208885  -1.996
## graduation_regunknown                  -0.273466   0.137186  -1.993
## employment_reghousewife/ househusband  -0.381103   0.153983  -2.475
## employment_regretired                  -0.009143   0.179871  -0.051
## employment_regnot employed             -0.104135   0.119271  -0.873
## employment_regunknown                  -0.610333   0.109929  -5.552
## number_of_baseline_comorbidities_reg1   0.033059   0.143903   0.230
## number_of_baseline_comorbidities_reg2   0.234064   0.157100   1.490
## number_of_baseline_comorbidities_reg3   0.241688   0.198619   1.217
## number_of_baseline_comorbidities_reg4   0.378625   0.350156   1.081
## number_of_baseline_comorbidities_regNA  0.131994   0.117885   1.120
##                                                Pr(>|z|)    
## (Intercept)                            0.00000002842640 ***
## studygroupCOVID-19                     0.00000000000113 ***
## age_years                                      0.001378 ** 
## BMI                                            0.646028    
## optechniqueSleeve                              0.000162 ***
## sex_regmale                                    0.000105 ***
## graduation_regintermediate                     0.072408 .  
## graduation_reglow                              0.210264    
## graduation_regwithout                          0.045943 *  
## graduation_regunknown                          0.046218 *  
## employment_reghousewife/ househusband          0.013325 *  
## employment_regretired                          0.959461    
## employment_regnot employed                     0.382614    
## employment_regunknown                  0.00000002822872 ***
## number_of_baseline_comorbidities_reg1          0.818302    
## number_of_baseline_comorbidities_reg2          0.136248    
## number_of_baseline_comorbidities_reg3          0.223663    
## number_of_baseline_comorbidities_reg4          0.279562    
## number_of_baseline_comorbidities_regNA         0.262849    
## ---
## Signif. codes:  0 '***' 0.001 '**' 0.01 '*' 0.05 '.' 0.1 ' ' 1
## 
## (Dispersion parameter for binomial family taken to be 1)
## 
##     Null deviance: 6009  on 5815  degrees of freedom
## Residual deviance: 5709  on 5797  degrees of freedom
##   (43 Beobachtungen als fehlend gelöscht)
## AIC: 5747
## 
## Number of Fisher Scoring iterations: 4
```

```
############################################################
##### Participation 1-year follow-up appointment ##########
############################################################

model_4 <- glm(fu2_completed ~ studygroup + age_years + BMI + optechnique  + sex_reg + graduation_reg + 
                    employment_reg + number_of_baseline_comorbidities_reg,
                    family = binomial,
                    data = STUDOQ_final)
              
summary(model_4)
```

```
## 
## Call:
## glm(formula = fu2_completed ~ studygroup + age_years + BMI + 
##     optechnique + sex_reg + graduation_reg + employment_reg + 
##     number_of_baseline_comorbidities_reg, family = binomial, 
##     data = STUDOQ_final)
## 
## Coefficients:
##                                          Estimate Std. Error z value
## (Intercept)                             0.9214214  0.2421019   3.806
## studygroupCOVID-19                     -0.3697898  0.0580867  -6.366
## age_years                               0.0106795  0.0026512   4.028
## BMI                                     0.0003733  0.0038371   0.097
## optechniqueSleeve                      -0.2491837  0.0622819  -4.001
## sex_regmale                            -0.3108945  0.0643254  -4.833
## graduation_regintermediate              0.1676255  0.1028973   1.629
## graduation_reglow                       0.2120853  0.1093871   1.939
## graduation_regwithout                   0.0225181  0.1863855   0.121
## graduation_regunknown                   0.1657868  0.1183014   1.401
## employment_reghousewife/ househusband  -0.3107570  0.1318670  -2.357
## employment_regretired                  -0.0015604  0.1434795  -0.011
## employment_regnot employed             -0.2667615  0.0963610  -2.768
## employment_regunknown                  -0.5138246  0.0981265  -5.236
## number_of_baseline_comorbidities_reg1  -0.0268204  0.1247342  -0.215
## number_of_baseline_comorbidities_reg2  -0.0112935  0.1326759  -0.085
## number_of_baseline_comorbidities_reg3  -0.1688710  0.1608704  -1.050
## number_of_baseline_comorbidities_reg4  -0.0011751  0.2730387  -0.004
## number_of_baseline_comorbidities_regNA -0.1938202  0.1030535  -1.881
##                                              Pr(>|z|)    
## (Intercept)                                  0.000141 ***
## studygroupCOVID-19                     0.000000000194 ***
## age_years                              0.000056216162 ***
## BMI                                          0.922497    
## optechniqueSleeve                      0.000063102628 ***
## sex_regmale                            0.000001343894 ***
## graduation_regintermediate                   0.103301    
## graduation_reglow                            0.052520 .  
## graduation_regwithout                        0.903838    
## graduation_regunknown                        0.161096    
## employment_reghousewife/ househusband        0.018443 *  
## employment_regretired                        0.991323    
## employment_regnot employed                   0.005634 ** 
## employment_regunknown                  0.000000163783 ***
## number_of_baseline_comorbidities_reg1        0.829751    
## number_of_baseline_comorbidities_reg2        0.932165    
## number_of_baseline_comorbidities_reg3        0.293841    
## number_of_baseline_comorbidities_reg4        0.996566    
## number_of_baseline_comorbidities_regNA       0.060003 .  
## ---
## Signif. codes:  0 '***' 0.001 '**' 0.01 '*' 0.05 '.' 0.1 ' ' 1
## 
## (Dispersion parameter for binomial family taken to be 1)
## 
##     Null deviance: 7297.7  on 5781  degrees of freedom
## Residual deviance: 7096.1  on 5763  degrees of freedom
##   (77 Beobachtungen als fehlend gelöscht)
## AIC: 7134.1
## 
## Number of Fisher Scoring iterations: 4
```

```
############################################################
##### Type-2 Diabetes at 1-year follow-up appointment  #####
############################################################

model_5 <- glm(diabetes_2_follow_up ~ studygroup + age_years + BMI + optechnique  + sex_reg + graduation_reg + 
                    employment_reg + number_of_baseline_comorbidities_reg,
                    family = binomial,
                    data = STUDOQ_final)
              
summary(model_5)
```

```
## 
## Call:
## glm(formula = diabetes_2_follow_up ~ studygroup + age_years + 
##     BMI + optechnique + sex_reg + graduation_reg + employment_reg + 
##     number_of_baseline_comorbidities_reg, family = binomial, 
##     data = STUDOQ_final)
## 
## Coefficients:
##                                         Estimate Std. Error z value
## (Intercept)                            -5.806857   0.533531 -10.884
## studygroupCOVID-19                      0.006319   0.090788   0.070
## age_years                               0.046788   0.004517  10.358
## BMI                                    -0.004108   0.006200  -0.663
## optechniqueSleeve                      -0.246933   0.095829  -2.577
## sex_regmale                             0.054164   0.101780   0.532
## graduation_regintermediate             -0.099638   0.171654  -0.580
## graduation_reglow                       0.070222   0.174326   0.403
## graduation_regwithout                   0.476908   0.284732   1.675
## graduation_regunknown                   0.348401   0.189331   1.840
## employment_reghousewife/ househusband  -0.019938   0.218975  -0.091
## employment_regretired                   0.176252   0.172738   1.020
## employment_regnot employed              0.515845   0.145106   3.555
## employment_regunknown                  -0.310148   0.155246  -1.998
## number_of_baseline_comorbidities_reg1   1.639294   0.410981   3.989
## number_of_baseline_comorbidities_reg2   2.302012   0.403182   5.710
## number_of_baseline_comorbidities_reg3   3.387617   0.410505   8.252
## number_of_baseline_comorbidities_reg4   5.023115   0.517175   9.713
## number_of_baseline_comorbidities_regNA  2.360516   0.390052   6.052
##                                                    Pr(>|z|)    
## (Intercept)                            < 0.0000000000000002 ***
## studygroupCOVID-19                                 0.944509    
## age_years                              < 0.0000000000000002 ***
## BMI                                                0.507619    
## optechniqueSleeve                                  0.009972 ** 
## sex_regmale                                        0.594613    
## graduation_regintermediate                         0.561604    
## graduation_reglow                                  0.687082    
## graduation_regwithout                              0.093947 .  
## graduation_regunknown                              0.065744 .  
## employment_reghousewife/ househusband              0.927451    
## employment_regretired                              0.307566    
## employment_regnot employed                         0.000378 ***
## employment_regunknown                              0.045740 *  
## number_of_baseline_comorbidities_reg1         0.00006642652 ***
## number_of_baseline_comorbidities_reg2         0.00000001132 ***
## number_of_baseline_comorbidities_reg3  < 0.0000000000000002 ***
## number_of_baseline_comorbidities_reg4  < 0.0000000000000002 ***
## number_of_baseline_comorbidities_regNA        0.00000000143 ***
## ---
## Signif. codes:  0 '***' 0.001 '**' 0.01 '*' 0.05 '.' 0.1 ' ' 1
## 
## (Dispersion parameter for binomial family taken to be 1)
## 
##     Null deviance: 3685.8  on 3891  degrees of freedom
## Residual deviance: 3141.2  on 3873  degrees of freedom
##   (1967 Beobachtungen als fehlend gelöscht)
## AIC: 3179.2
## 
## Number of Fisher Scoring iterations: 6
```

```
############################################################
##### Hypertension at 1-year follow-up appointment  ########
############################################################

model_6 <- glm(hypertension_follow_up ~ studygroup + age_years + BMI + optechnique  + sex_reg + graduation_reg + 
                    employment_reg + number_of_baseline_comorbidities_reg,
                    family = binomial,
                    data = STUDOQ_final)
              
summary(model_6)
```

```
## 
## Call:
## glm(formula = hypertension_follow_up ~ studygroup + age_years + 
##     BMI + optechnique + sex_reg + graduation_reg + employment_reg + 
##     number_of_baseline_comorbidities_reg, family = binomial, 
##     data = STUDOQ_final)
## 
## Coefficients:
##                                         Estimate Std. Error z value
## (Intercept)                            -7.171498   0.399568 -17.948
## studygroupCOVID-19                     -0.112987   0.073795  -1.531
## age_years                               0.062106   0.003698  16.792
## BMI                                     0.032456   0.005005   6.484
## optechniqueSleeve                      -0.063294   0.078453  -0.807
## sex_regmale                             0.240424   0.083780   2.870
## graduation_regintermediate              0.026482   0.133692   0.198
## graduation_reglow                      -0.246243   0.141267  -1.743
## graduation_regwithout                  -0.420976   0.256019  -1.644
## graduation_regunknown                   0.407113   0.153395   2.654
## employment_reghousewife/ househusband  -0.073026   0.174466  -0.419
## employment_regretired                   0.277294   0.161916   1.713
## employment_regnot employed              0.184611   0.125559   1.470
## employment_regunknown                  -0.328670   0.125957  -2.609
## number_of_baseline_comorbidities_reg1   2.569473   0.261049   9.843
## number_of_baseline_comorbidities_reg2   3.094052   0.265333  11.661
## number_of_baseline_comorbidities_reg3   3.067602   0.287421  10.673
## number_of_baseline_comorbidities_reg4   3.442719   0.397008   8.672
## number_of_baseline_comorbidities_regNA  2.335851   0.250509   9.324
##                                                    Pr(>|z|)    
## (Intercept)                            < 0.0000000000000002 ***
## studygroupCOVID-19                                  0.12575    
## age_years                              < 0.0000000000000002 ***
## BMI                                         0.0000000000892 ***
## optechniqueSleeve                                   0.41979    
## sex_regmale                                         0.00411 ** 
## graduation_regintermediate                          0.84298    
## graduation_reglow                                   0.08132 .  
## graduation_regwithout                               0.10011    
## graduation_regunknown                               0.00795 ** 
## employment_reghousewife/ househusband               0.67553    
## employment_regretired                               0.08679 .  
## employment_regnot employed                          0.14148    
## employment_regunknown                               0.00907 ** 
## number_of_baseline_comorbidities_reg1  < 0.0000000000000002 ***
## number_of_baseline_comorbidities_reg2  < 0.0000000000000002 ***
## number_of_baseline_comorbidities_reg3  < 0.0000000000000002 ***
## number_of_baseline_comorbidities_reg4  < 0.0000000000000002 ***
## number_of_baseline_comorbidities_regNA < 0.0000000000000002 ***
## ---
## Signif. codes:  0 '***' 0.001 '**' 0.01 '*' 0.05 '.' 0.1 ' ' 1
## 
## (Dispersion parameter for binomial family taken to be 1)
## 
##     Null deviance: 5239.7  on 3891  degrees of freedom
## Residual deviance: 4344.9  on 3873  degrees of freedom
##   (1967 Beobachtungen als fehlend gelöscht)
## AIC: 4382.9
## 
## Number of Fisher Scoring iterations: 5
```

```
#########################################################
######## Store the results in table_3 ###################
#########################################################

### Set up dataframe to store results

table_3 <- data.frame(    outcome = c("%TWL", "BQL", "3-month fu participation", "1-year fu participation", "Type-2 Diabetes", "Hypertension"),
                          N = c(rep(0, 6)),
                          Model_type = c(rep(0, 6)),
                          Estimate = c(rep(0, 6)), 
                          CI = c(rep(0, 6)),
                          p_value = c(rep(0, 6)))


#####################
##### %TWL ##########
#####################

table_3[1,2] <- nobs(model_1)
table_3[1,3] <- "linear"
table_3[1,4] <- round(model_1$coefficients[2], digits = 3)

summary_1 <- summary(model_1)
# Calculate CI
conf_1 <- confint(model_1, level=0.95)
# Extract the upper and lower bounds of the confidence interval 
lower_ci_1 <- round(conf_1[2, 1], digits = 2)
upper_ci_1 <- round(conf_1[2, 2], digits = 2)
CI_1 <- paste("[", lower_ci_1, ", ", upper_ci_1,"]", sep="" )
table_3[1,5] <- CI_1

table_3[1,6] <- round(summary_1$coefficients[2,4], digits = 3)

####################
##### BQL ##########
####################

table_3[2,2] <- nobs(model_2)
table_3[2,3] <- "Linear"
table_3[2,4] <- round(model_2$coefficients[2], digits = 3)

summary_2 <- summary(model_2)
# Calculate CI
conf_2 <- confint(model_2, level=0.95)
# Extract the upper and lower bounds of the confidence interval 
lower_ci_2 <- round(conf_2[2, 1], digits = 2)
upper_ci_2 <- round(conf_2[2, 2], digits = 2)
CI_2 <- paste("[", lower_ci_2, ", ", upper_ci_2,"]", sep="" )
table_3[2,5] <- CI_2

table_3[2,6] <- round(summary_2$coefficients[2,4], digits = 3)


############################################################
##### Participation 3-month follow-up appointment ##########
############################################################

table_3[3,2] <- nobs(model_3)

table_3[3,3] <- "Logistic"
OR_3 <- round(exp(model_3$coefficients[2]), digits = 3)
table_3[3,4] <- OR_3

summary_3 <- summary(model_3)
# Calculate CI
conf_3 <- confint(model_3, level=0.95)
# Calculate exponential values of confidence intervals (for OR)
exp_conf_3 <- exp(conf_3)
# Extract the upper and lower bounds of the confidence interval
lower_ci_3 <- round(exp_conf_3[2, 1], digits = 2)
upper_ci_3 <- round(exp_conf_3[2, 2], digits = 2)
CI_3 <- paste("[", lower_ci_3, ", ", upper_ci_3,"]", sep="" )
table_3[3,5] <- CI_3

table_3[3,6] <- round(summary_3$coefficients[2,4], digits = 3)

############################################################
##### Participation 1-year follow-up appointment ##########
############################################################


table_3[4,2] <- nobs(model_4)

table_3[4,3] <- "Logistic"
OR_4 <- round(exp(model_4$coefficients[2]), digits = 3)
table_3[4,4] <- OR_4

summary_4 <- summary(model_4)
# Calculate CI
conf_4 <- confint(model_4, level=0.95)
# Calculate exponential values of confidence intervals (for OR)
exp_conf_4 <- exp(conf_4)
# Extract the upper and lower bounds of the confidence interval
lower_ci_4 <- round(exp_conf_4[2, 1], digits = 2)
upper_ci_4 <- round(exp_conf_4[2, 2], digits = 2)
CI_4 <- paste("[", lower_ci_4, ", ", upper_ci_4,"]", sep="" )
table_3[4,5] <- CI_4

table_3[4,6] <- round(summary_4$coefficients[2,4], digits = 3)

############################################################
##### Type-2 Diabetes at 1-year follow-up appointment  #####
############################################################

table_3[5,2] <- nobs(model_5)

table_3[5,3] <- "Logistic"
OR_5 <- round(exp(model_5$coefficients[2]), digits = 3)
table_3[5,4] <- OR_5

summary_5 <- summary(model_5)
# Calculate CI
conf_5 <- confint(model_5, level=0.95)
# Calculate exponential values of confidence intervals (for OR)
exp_conf_5 <- exp(conf_5)
# Extract the upper and lower bounds of the confidence interval
lower_ci_5 <- round(exp_conf_5[2, 1], digits = 2)
upper_ci_5 <- round(exp_conf_5[2, 2], digits = 2)
CI_5 <- paste("[", lower_ci_5, ", ", upper_ci_5,"]", sep="" )
table_3[5,5] <- CI_5

table_3[5,6] <- round(summary_5$coefficients[2,4], digits = 3)

############################################################
##### Hypertension at 1-year follow-up appointment  ########
############################################################


table_3[6,2] <- nobs(model_6)

table_3[6,3] <- "Logistic"
OR_6 <- round(exp(model_6$coefficients[2]), digits = 3)
table_3[6,4] <- OR_6

summary_6 <- summary(model_6)
# Calculate CI
conf_6 <- confint(model_6, level=0.95)
# Calculate exponential values of confidence intervals (for OR)
exp_conf_6 <- exp(conf_6)
# Extract the upper and lower bounds of the confidence interval
lower_ci_6 <- round(exp_conf_6[2, 1], digits = 2)
upper_ci_6 <- round(exp_conf_6[2, 2], digits = 2)
CI_6 <- paste("[", lower_ci_6, ", ", upper_ci_6,"]", sep="" )
table_3[6,5] <- CI_6

table_3[6,6] <- round(summary_6$coefficients[2,4], digits = 3)


#### Check out table_3

table_3
```

```
##                    outcome    N Model_type Estimate             CI p_value
## 1                     %TWL 3901     linear    0.287  [-0.32, 0.89]   0.353
## 2                      BQL 1218     Linear   -0.140 [-0.21, -0.07]   0.000
## 3 3-month fu participation 5816   Logistic    0.617    [0.54, 0.7]   0.000
## 4  1-year fu participation 5782   Logistic    0.691   [0.62, 0.77]   0.000
## 5          Type-2 Diabetes 3892   Logistic    1.006    [0.84, 1.2]   0.945
## 6             Hypertension 3892   Logistic    0.893   [0.77, 1.03]   0.126
```

```
### Output input table
write.xlsx(table_3, "Table_3_R_input.xlsx")
```

## 4.3 Supplementary Material

### 4.3.1 Table S1

```
# Create dataframe to store the results

NA_table <- data.frame(variable = c("sex", "age", "graduation", "employment", "weight", "BMI", "OP technique", "diabetes type 2 at baseline", "hypertension at baseline", "dyslipidemia at baseline", "sleep_apnoea at baseline", "number of comorbidities at baseline",  "completed 3-month fu", "completed 1-year fu","% TWL", "BQL", "diabetes type 2 at fu", "hypertension at fu"), overall = c(rep(0, 18)), COVID_19 = c(rep(0, 18)), "No_COVID_19" = c(rep(0, 18)))

variable_list <- c("sex", "age_years", "graduation", "employment", "weight_baseline" , "BMI", "optechnique", "diabetes_2_baseline", "hypertension_baseline" , "dyslipidemia_baseline", "sleep_apnoea_baseline", "number_of_baseline_comorbidities", "fu1_completed", "fu2_completed", "total_weight_loss_percentage", "BQL", "diabetes_2_follow_up", "hypertension_follow_up")

# Define total number of observations

n <- nrow(STUDOQ_final)
n_COVID_19 <- nrow(STUDOQ[STUDOQ$studygroup == "COVID-19",])
n_No_COVID_19 <- nrow(STUDOQ[STUDOQ$studygroup == "No-COVID-19",]) 

# Fill the datframe for the overall group

for (i in 1:length(variable_list)) {
   
  missing_percentage <- sum(is.na(STUDOQ[[variable_list[i]]])) / n    
  
  NA_table[i, 2] <- missing_percentage 
  
}


# Fill the datframe for the COVID-19 group

STUDOQ_COVID_19_group <- filter(STUDOQ, studygroup == "COVID-19")
  
  
for (i in 1:length(variable_list)) {
   
  missing_percentage <- sum(is.na(STUDOQ_COVID_19_group[[variable_list[i]]])) / n_COVID_19    
  
  NA_table[i, 3] <- missing_percentage 
  
}


# Fill the datframe for the COVID-19 group

STUDOQ_No_COVID_19_group <- filter(STUDOQ, studygroup == "No-COVID-19")
  
  
for (i in 1:length(variable_list)) {
   
  missing_percentage <- sum(is.na(STUDOQ_No_COVID_19_group[[variable_list[i]]])) / n_No_COVID_19    
  
  NA_table[i, 4] <- missing_percentage 
  
}

# Check out the table

table_S1 <- NA_table

table_S1
```

```
##                               variable      overall    COVID_19  No_COVID_19
## 1                                  sex 0.0003413552 0.000312989 0.0003753754
## 2                                  age 0.0000000000 0.000000000 0.0000000000
## 3                           graduation 0.2462877624 0.237871674 0.2563813814
## 4                           employment 0.2454343745 0.236619718 0.2560060060
## 5                               weight 0.0037549070 0.000000000 0.0082582583
## 6                                  BMI 0.0037549070 0.000000000 0.0082582583
## 7                         OP technique 0.0000000000 0.000000000 0.0000000000
## 8          diabetes type 2 at baseline 0.5953234340 0.619405321 0.5664414414
## 9             hypertension at baseline 0.5953234340 0.619405321 0.5664414414
## 10            dyslipidemia at baseline 0.5953234340 0.619405321 0.5664414414
## 11            sleep_apnoea at baseline 0.5953234340 0.619405321 0.5664414414
## 12 number of comorbidities at baseline 0.5953234340 0.619405321 0.5664414414
## 13                completed 3-month fu 0.0035842294 0.005320814 0.0015015015
## 14                 completed 1-year fu 0.0093872675 0.012832551 0.0052552553
## 15                               % TWL 0.3341867213 0.371205008 0.2897897898
## 16                                 BQL 0.7921146953 0.801877934 0.7804054054
## 17               diabetes type 2 at fu 0.3357228196 0.370892019 0.2935435435
## 18                  hypertension at fu 0.3357228196 0.370892019 0.2935435435
```

```
# Create Excel Table that serves as Input file for creating Table A1 of the paper

write.xlsx(table_S1, "Table_S1_R_input.xlsx")
```

### 4.3.2 Numbers for Flowchart (Supplementary Material 6)

```
### Eligible patients
nrow(STUDOQ_final)
```

```
## [1] 5859
```

```
### Patients with data for %TWL
# total
nrow(filter(STUDOQ_final, is.na(total_weight_loss_percentage)==FALSE))
```

```
## [1] 3901
```

```
# No Covid-19
nrow(filter(STUDOQ_final_NO_COVID_19, is.na(total_weight_loss_percentage)==FALSE))
```

```
## [1] 1892
```

```
#Covid-19
nrow(filter(STUDOQ_final_COVID_19, is.na(total_weight_loss_percentage)==FALSE))
```

```
## [1] 2009
```

```
### Patients with data for BQL
# total
nrow(filter(STUDOQ_final, is.na(BQL)==FALSE))
```

```
## [1] 1218
```

```
# No Covid-19
nrow(filter(STUDOQ_final_NO_COVID_19, is.na(BQL)==FALSE))
```

```
## [1] 585
```

```
#Covid-19
nrow(filter(STUDOQ_final_COVID_19, is.na(BQL)==FALSE))
```

```
## [1] 633
```

```
### Patients with data for participation in 3-month fu
# total
nrow(filter(STUDOQ_final, is.na(fu1_completed)==FALSE))
```

```
## [1] 5838
```

```
# No Covid-19
nrow(filter(STUDOQ_final_NO_COVID_19, is.na(fu1_completed)==FALSE))
```

```
## [1] 2660
```

```
#Covid-19
nrow(filter(STUDOQ_final_COVID_19, is.na(fu1_completed)==FALSE))
```

```
## [1] 3178
```

```
### Patients with data for participation in 1-year fu
# total
nrow(filter(STUDOQ_final, is.na(fu2_completed)==FALSE))
```

```
## [1] 5804
```

```
# No Covid-19
nrow(filter(STUDOQ_final_NO_COVID_19, is.na(fu2_completed)==FALSE))
```

```
## [1] 2650
```

```
#Covid-19
nrow(filter(STUDOQ_final_COVID_19, is.na(fu2_completed)==FALSE))
```

```
## [1] 3154
```

```
### Patients with data for T2D at fu
# total
nrow(filter(STUDOQ_final, is.na(diabetes_2_follow_up)==FALSE))
```

```
## [1] 3892
```

```
# No Covid-19
nrow(filter(STUDOQ_final_NO_COVID_19, is.na(diabetes_2_follow_up)==FALSE))
```

```
## [1] 1882
```

```
#Covid-19
nrow(filter(STUDOQ_final_COVID_19, is.na(diabetes_2_follow_up)==FALSE))
```

```
## [1] 2010
```

```
### Patients with data for hypertension at fu
# total
nrow(filter(STUDOQ_final, is.na(hypertension_follow_up)==FALSE))
```

```
## [1] 3892
```

```
# No Covid-19
nrow(filter(STUDOQ_final_NO_COVID_19, is.na(hypertension_follow_up)==FALSE))
```

```
## [1] 1882
```

```
#Covid-19
nrow(filter(STUDOQ_final_COVID_19, is.na(hypertension_follow_up)==FALSE))
```

```
## [1] 2010
```
